# Insight into the phytochemical profile and antimicrobial activities of *Amomum subulatum* and *Amomum xanthioides*: an *in vitro* and *in silico* study

**DOI:** 10.3389/fpls.2023.1136961

**Published:** 2023-04-20

**Authors:** Mohammed H. Alruhaili, Mohammed S. Almuhayawi, Hattan S. Gattan, Mohanned Talal Alharbi, Mohammed K. Nagshabandi, Soad K. Al Jaouni, Samy Selim, Hamada AbdElgawad

**Affiliations:** ^1^ Department of Clinical Microbiology and Immunology Faculty of Medicine, King AbdulAziz University, Jeddah, Saudi Arabia; ^2^ Special Infectious Agents Unit, King Fahad Medical Research Center, King AbdulAziz University, Jeddah, Saudi Arabia; ^3^ Department of Medical Laboratory Sciences, Faculty of Applied Medical Sciences, King Abdulaziz University, Jeddah, Saudi Arabia; ^4^ Department of Medical Microbiology and Parasitology, Faculty of Medicine, University of Jeddah, Jeddah, Saudi Arabia; ^5^ Department of Hematology/Oncology, Yousef Abdulatif Jameel Scientific Chair of Prophetic Medicine Application, Faculty of Medicine, King Abdulaziz University, Jeddah, Saudi Arabia; ^6^ Department of Clinical Laboratory Sciences, College of Applied Medical Sciences, Jouf University, Sakaka, Saudi Arabia; ^7^ Department of Botany and Microbiology, Faculty of Science, Beni-Suef University, Beni-Suef, Egypt

**Keywords:** phytochemical analysis, natural products, medicinal plants, *Amomum subulatom*, *Amomum xanthioides*, antioxidant activity, antimicrobial activities, molecular docking

## Abstract

**Introduction:**

Medicinal plants have been considered as potential source of therapeutics or as starting materials in drugs formulation.

**Methods:**

The current study aims to shed light on the therapeutic potential of the *Amomum subulatom* and *Amomum xanthioides* Fruits by analyzing the phytochemical composition of their seeds and fruits using gas chromatography-mass spectrometry (GC-MS) and high-performance liquid chromatography (HPLC) techniques to determine the presence of bioactive components such as flavonoids, phenols, vitamins, steroids, and essential oils.

**Results and Discussion:**

The protein content is usually higher than the total lipids in both species except the fruit of *A. subulatum* which contain more lipids than proteins. The total protein contents for *A. subulatum* were 235.03 ± 21.49 and 227.49 ± 25.82 mg/g dry weight while for *A. xanthioides* were 201.9 ± 37.79 and 294.99 ± 37.93 mg/g dry weight for seeds and fruit, respectively. The Carvacrol levels in *A. subulatum* is 20 times higher than that in *A. xanthioides*. Lower levels of α-Thujene, Phyllanderenes, Ascaridole, and Pinocarvone were also observed in both species. According to DPPH (2,2-diphenylpicrylhydrazyl) assay, seed the extract of *A. subulatum* exhibited the highest antioxidant activity (78.26±9.27 %) followed by the seed extract of *A. xanthioides* (68.21±2.56 %). Similarly, FRAP (Ferric Reducing Antioxidant Power) assay showed that the highest antioxidant activity was exhibited by the seed extract of the two species; 20.14±1.11 and 21.18±1.04 µmol trolox g−1 DW for *A. subulatum* and *A. xanthioides*, respectively. In terms of anti-lipid peroxidation, relatively higher values were obtained for the fruit extract of *A. subulatum* (6.08±0.35) and the seed extract of *A. xanthioides* (6.11±0.55). Ethanolic seed extracts of *A. subulatum* had the highest efficiency against four Gram-negative bacterial species which causes serious human diseases, namely *Pseudomonas aeruginosa*, *Proteus vulgaris*, *Enterobacter aerogenes*, and *Salmonella typhimurium*. In addition, *P. aeruginosa* was also inhibited by the fruit extract of both *A. subulatum* and *A. xanthioides*. For the seed extract of *A. xanthioides*, large inhibition zones were formed against *P. vulgaris* and the fungus *Candida albicans*. Finally, we have *in silico* explored the mode of action of these plants by performing detailed molecular modeling studies and showed that the antimicrobial activities of these plants could be attributed to the high binding affinity of their bioactive compounds to bind to the active sites of the sterol 14-alpha demethylase and the transcriptional regulator MvfR.

**Conclusion:**

These findings demonstrate the two species extracts possess high biological activities and therapeutical values, which increases their potential value in a number of therapeutic applications.

## 1 Introduction

Historically, medicinal plants were used worldwide either directly as therapeutics or as starting materials in drug formulation (Grover et al., 2002; Bauer and Brönstrup, 2014; Pešić and Stanković, 2015). The World Health Organization (WHO) evaluates that approximately 80% of the people worldwide now depend on medicinal plants for basic medical requirements (WHO establishes the Global Centre for Traditional Medicine in India (n.d.)). Medicinal plants are used to treat numerous illnesses, including diabetes, cardiovascular diseases, nervous system disorders, asthma, hypertension, and cancer (Prasathkumar et al., 2021). Natural antimicrobials found in medicinal plants are highly effective against newly emerging microbial strains (Cowan, 1999; Tijjani et al., 2011). Due to the inappropriate and excessive use of antimicrobial medications, microbes have become resistant to many antibiotics, which poses a significant challenge for the treatment of infectious illnesses (Davies, 1994; McGregor et al., 2014; Saleem et al., 2019; Thompson, 2022). Antibiotic resistance occurs due to the constant generation of resistant strains to drugs and the adaptation of microorganisms to frequently used antibiotics (Ventola, 2015; Aslam et al., 2018; Saleem et al., 2019). Lately, the interest in medicinal plants has increased because of the great potential of plant-based medicines. Consequently, one of the main sources of commercial pharmaceuticals is still the useful compounds extracted from medicinal plants. In addition to being medicinal, numerous plant extracts have been widely employed in fragrances, food flavoring, and food preservation (Pandey et al., 2017; Delesa, 2018).

Medicinal plants are distributed and considered a valuable source of novel medications worldwide (Balunas and Kinghorn, 2005; Rafieian-Kopaei, 2012). The usage of medicinal plants is expanding quickly globally due to the rising need for herbal medicines, healthcare products, and plant secondary metabolites. Therefore, isolation and purification of extracts from medicinal plants are important for discovering and developing new drugs. *A. subulatum*, generally recognized as black cardamom, is a herbaceous plant used as a medicinal spice in India (Gautam et al., 2016). It has historically been used to treat vomiting, abdominal pain, gastrointestinal (GI) infections, and rectal diseases (Kumar et al., 2015; Alam and Singh, 2021; Dhakal et al., 2022). The therapeutic capabilities of this medicinal herb have gained great attention in the past years since it is found to be a source of antimicrobials, antioxidants, anti-inflammatory, and cardio-protective compounds (Bhaswant et al., 2015; Gautam et al., 2016). The essential oil components of *A. subulatum* are primarily responsible for its therapeutic capability (Dhakal et al., 2022). Essential oils from *A. subulatum* seeds are found to have an antimicrobial effect against various pathogens including *Staphylococcus aureus*, *Escherichia coli*, *Bacillus pumilus*, *Pseudomonas aeruginosa*, and *Aspergillus niger* (Agnihotri and Wakode, 2010; Satyal et al., 2012). Hexane seed extract of *A. subulatum* exhibited high cellular toxicity on HeLa and MCF-7 cell lines, which are human cancer cell lines, indicating its anticancer potential, while the ethyl acetate extract possessed a considerable antioxidant activity based on the free radical scavenging assay (Sharma et al., 2017). In addition, fruit extracts of *A. subulatum* have shown high anti-inflammatory effects in rats with carrageenan-induced paw edema when compared with diclofenac, the main drug for this disease (Alam et al., 2011). The fruit extracts also exhibited anticancer activity in mice through the control of cytokines that cause inflammation as well as the NF-κB signaling (Sudarsanan et al., 2021). A recent study discovered that the dichloromethane extract of *A. subulatum* had an apoptotic effect against lung cancer cells (Makhija et al., 2022).

*Amomum xanthioides* is a vigorous herb that is used for food and medicinal purposes in southern China, India, Thailand, Vietnam, Laos, and Cambodia (Lamxay and Newman, 2012). Ethyl acetate extracts from *A. xanthioides* seeds are found to have therapeutic potential against liver fibrosis in rat models (Kim et al., 2015). When administered to high-fat-diet mice, ethyl acetate extract from *A. xanthioides* showed an anti-fatty liver effect, indicating its therapeutic potential in managing non-alcoholic fatty liver disorders (Im et al., 2020). Moreover, anti-inflammatory effects are associated with *A. xanthioides* extracts in atopic dermatitis, a chronic relapsing skin inflammation (Choi et al., 2017). Essential oils extracted from *A. xanthioides* fruits were found to have growth inhibitory effects on *Enterococcus faecalis*, *Bacillus cereus*, *P. aeruginosa*, and *S. aureus*, with minimum inhibitory concentration values between 100 and 200 g/ml (Thinh et al., 2022).

In spite of all the previous data, *A. subulatum* and *A. xanthioides* are not being given much consideration for therapeutic development as a result of a lack of comprehensive chemical analysis and pharmacological research (Thinh et al., 2021; Dhakal et al., 2022). In this manner, the current study aimed to provide insight into the phytochemical composition of both species by investigating the bioactive components in their seeds and fruits, including flavonoids, phenols, vitamins, steroids, and essential oils. To this end, techniques such as gas chromatography–mass spectrometry (GC-MS) and high-performance liquid chromatography (HPLC) were employed. In addition, the antimicrobial and antioxidant potentials of the ethanol extracts were assessed through multiple assays. Finally, computational docking was performed to investigate the molecular interactions of the proposed compounds against certain microbial proteins determined from prior analysis to confirm the antimicrobial activity observed from these compounds.

## 2 Materials and methods

### 2.1 Plant sample collection and identification

The seeds and fruits of *A. subulatum* and *A. xanthioides* were obtained at a local Pakistani store in Antwerp, Belgium. The plant specimen was identified and authenticated by a plant taxonomist at the Botany Department of Beni Suef University, Egypt.

### 2.2 Total nutrients

Sugars were extracted in 0.2 g dry weight of *Amomum* seeds and fruit samples in 2 ml of boiled distilled water for 60 min at 100°C. After being cooled, the extract was centrifuged at 6,000 ×*g* for 15 min. The pellet was re-extracted using 2 ml of boiled distilled water. After centrifugation, the two supernatants were combined for further analysis. The concentration of total sugars in the supernatant was assessed following Nelson’s method as defined by Clark and Switzer (1977). Sugar extract was added to freshly prepared Nelson’s alkaline copper reagent (1:1 v/v), and the mixture was boiled for 20 min. Subsequently, 1 ml of arseno-molybdate reagent was added to the reaction mixture with shaking to dissolve Cu_2_O. When the effervescence stopped, the change in color intensity was measured at 540 nm. The reduced sugar content was determined by means of a glucose standard curve. The protein concentration was assessed for *Amomum* seeds and fruits (0.2 g DW) extracted twice in NaOH (0.4% w/v) at room temperature. Extracts were shaken by using an orbital shaker at 220 rpm for 45 min. The total protein content was measured by using Lowry’s method at 660 nm (Oh et al., 1951). Bovine serum albumin was used as a standard reagent. Total lipid concentrations were determined, where *Amomum* seeds and fruit samples were homogenized twice in a 2:1 mixture of chloroform/methanol (v/v). Then plants were centrifuged for 15 min at 3,000 ×*g*. In a 4:1 ratio of toluene:ethanol (v/v), the pellets were re-dissolved. The total lipid content was determined after concentration. Gravimetric analysis was used to determine the extracted lipids, which were represented as weight (g) per fresh weight (g) of the plant. To eliminate unwanted protein and starch, crude fibers were gelatinized by a heat-stable alpha-amylase at pH 6 and 100°C for 25 min and then enzymatically digested by a combination of protease (pH 7.5, 60°C, 25 min) and amyloglucosidase (pH 6, 0°C, 30 min). Fibers were allowed to precipitate in ethanol for washing, and the residues were weighed after washing.

### 2.3 Mineral quantification

The recognition of mineral elements was achieved according to AbdElgawad et al. (2014), where 200 mg dry weight of *Amomum* seeds and fruit samples was digested in 5:1 (v:v) HNO_3_/H_2_O solution for 30 min. Thereafter, macroelements and microelements were estimated (inductively coupled plasma–mass spectrometry (ICP-MS), Finnigan Element XR, and Scientific, Bremen, Germany). Nitric acid (1%) was used as blank.

### 2.4 Total phenolics and flavonoids

Total polyphenols and flavonoids were obtained by homogenizing 200 mg dry weight of *Amomum* seeds and fruit samples in 2 ml of 80% ethanol (v/v). The contents of phenolic and flavonoid were assessed using the Folin–Ciocalteu and aluminum chloride colorimetric assays, respectively (Saleh and Madany, 2015), with gallic acid and quercetin as standards, respectively (Sigma-Aldrich Co., St. Louis, MO, USA). For the determination of total phenolics, 1 ml of the phenolic extract was mixed with 1 ml of 10% Folin–Ciocalteu phenol reagent and 1 ml of 20% anhydrous Na_2_CO_3_ and afterward filled to a given volume with distilled water. The absorbance of the resulting blue color was observed after 30 min at 650 nm against a water-reagent blank (samples extracted and replaced by distilled water). The total phenolic content was determined from a catechol (Sigma-Aldrich Co., St. Louis, MO, USA) standard curve and reported as mg gallic/g dry weight. Total flavonoid content was determined by mixing 0.25 ml of the extract with 1.25 ml of distilled water in a test tube, followed by the addition of 75 µl of 5% (w/v) Na nitrite solution. After 6 min, 150 µl of 10% (w/v) AlCl_3_ was added, and the mixture was left for another 5 min prior to adding 0.5 ml of 1 M of sodium hydroxide. The solution was filled and mixed with distilled water up to 2.5 ml, and the absorbance was recorded at 510 nm. The total flavonoid content was measured using a quercetin standard curve and reported as mg quercetin/g dry weight.

### 2.5 Total alkaloid estimation

The total alkaloid was estimated following the protocol described by Sreevidya et al (Sreevidya and Mehrotra, 2003). along with the bismuth nitrate pentahydrate (Bi(NO_3_)_3_·5H_2_O) calibration curve. Samples were extracted in methanol-HCl at pH 2–2.5. The extract was mixed with Dragendorff’s reagent (bismuth nitrate pentahydrate, glacial acetic acid, and 8.0 g of potassium iodide) and centrifuged for 10 min at 5,000 rpm, 25°C. The precipitate was further washed twice with methanol, and the residue was added to a disodium sulfide solution. The resulting brownish-black precipitate was afterward centrifuged for 10 min at 5,000 rpm. The residue was dissolved in concentrated nitric acid by mild warming. This solution was diluted with distilled water and mixed with a 3% thiourea solution. At 435 nm, the absorbance was recorded in comparison to a blank containing HNO_3_ and thiourea by using a spectrophotometer (Perkin Elmer Lambda 25).

### 2.6 Saponins

The extraction and quantification of saponin in *Amomum* seeds and fruit samples were performed (Lai et al., 2013). Ground dried seeds and fruits were extracted in petroleum ether and shaken for 4 h at 1,000 rpm and 25°C ± 3°C. The solvent was then removed using a vacuum rotary evaporator at 60°C. Saponin in residues was extracted in 80% aqueous methanol and shaken for another 4 h. The extract was filtered and preserved at 4°C in the dark. For quantification, the spectrophotometric technique was applied to estimate the total saponin content of the samples. In a cold-water bath (0°C), 0.1 ml of the extract attained above was added to 0.4 ml of methanol solution (80%), 0.5 ml of freshly prepared vanillin solution (8% (w/v; prepared in ethanol), and 5.0 ml of sulfuric acid (72%). After that, the mixture was put in a water bath at 60°C for 10 min and lastly cooled in ice-cold water. The absorbance was measured at 544 nm against a reagent blank with a UV–Vis spectrophotometer (Shimadzu UV-160A PC, Shimadzu Corporation, Kyoto, Japan). The reagent blank was prepared by applying the same technique, but the extract was exchanged with an equivalent volume of 80% methanol. The results were estimated from a standard curve plotted with various crude soya saponin concentrations (0, 1,000, 2,000, and 3,000 ppm) containing at least 80% saponin (Waki, Osaki, Japan) in 80% aqueous methanol and expressed as mg soya saponin/100 g sample.

### 2.7 Fatty acid profile

The fatty acid profile was quantified according to Hassan et al. (2018). To obtain lipophilic fraction, 0.2 g dry weight of *Amomum* seeds and fruit samples was extracted in chloroform/methanol (2:1, v/v) at 25°C in the presence of the internal standard tripentadecanoic acid triglyceride. Fatty acids were derivatized with 1% pentafluorobenzyl (PFB) bromide in acetonitrile at room temperature for 20 min. The fatty acid PFB esters dissolved in 50 μl of *iso*-octane are injected. The investigation was applied by means of an Agilent single-quadrupole mass spectrometer with an inert mass selective detector (MSD-5975C detector, Agilent Technologies, Santa Clara, CA, USA) coupled directly to an Agilent 7890A gas chromatograph that was equipped with a split–splitless injector, a quick-swap assembly, an Agilent model 7693 autosampler, and an HP-5MS fused silica capillary column (5% phenyl/95% dimethylpolysiloxane, 30 m × 0.25 mm i.d., film thickness 0.25 μm, Agilent Technologies, USA). The temperature of the oven was kept at 80°C for 2 min and then elevated up to 200°C at 5°C/min (1 min hold) and then to 280°C at 20°C/min (3 min hold). A 1.0-μl sample was injected by means of a split mode (split ratio, 1:10). At a flow rate of 1.5 ml/min, helium gas was employed as a carrier gas. For MS detection, an electron ionization technique with an ionization energy of 70 eV was applied. The temperatures of the injector and MS transfer line were set at 220°C and 290°C, respectively. The mass scan ranged from 50 to 550 *m*/*z* with an E_m_ voltage of 1,035 V. The quantitative examination of fatty acids is performed by comparing the target molecule’s mass spectrometric ion signal to that of an equivalent standard. Fatty acid standard curves were obtained by serially diluting a standard mixture of unlabeled quantitative fatty acid standards at specified quantities. Each fatty acid is given in quantitative standard dilution sets in the 0.15–500-ng range. A standard curve is created by doing a linear regression model on the ratio of the quantitative standard and internal standard ion yields plotted *vs.* the quantitative standard absolute quantities. The fatty acid content of the sample is then determined from the standard curve using analyte/internal standard ion yield ratios. In parallel, software freely available for the deconvolution of fatty acids spectrum was applied; i.e., the metabolite libraries available for metabolite identification were obtained using NIST08, a generalized chemical library (http://chemdata.nist.gov/mass-spc/ms-search/), and those specifically for metabolites were obtained using the Golm Metabolome (http://gmd.mpimp-golm.mpg.de).

### 2.8 Essential oil analysis

Plant samples were placed in 1 L of distilled water and subjected to hydrodistillation for 3 h, using a Clevenger-type apparatus (1.5% yield). To remove water remnants from essential oil, anhydrous Na_2_SO_4_ was utilized. The essential oil was stored at +4°C until tested and analyzed.

### 2.9 Gas chromatography and gas chromatography–mass spectrometry

Gas chromatography–flame ionization detector (GC-FID) and GC-MS were used to determine the essential oil both quantitatively and qualitatively. GC analyses were performed on a Varian (Les Ulis, France) Star 3400 Cx chromatograph fitted with a fused silica capillary DB-5MS (5% phenyl methylpolysiloxane; 30 m/0.25 mm; film thickness, 0.25 mm) column. Chromatographic conditions were 60°C to 260°C temperature increases with a gradient of 5°C/min and 15 min isothermal at 260°C. A second increase was applied, reaching 340°C at 40°C/min. The total time of the analysis was 57 min. Petroleum ether was used for dissolving the oils to avoid saturating the column. Injection of the sample was performed at a split mode ratio of 1:10. Helium (purity 99.999%) was utilized as the carrier gas at 1 ml/min. The injector was operated at 200°C. The mass spectrometer (Varian Saturn GC/MS/MS 4D) was set at an electron multiplier voltage between 1,400 and 1,500 V and an emission current of 10 mA. The transfer line’s temperature was 170°C, whereas the trap’s temperature was 150°C. A total of 40 to 650 atomic mass units was covered by the mass scanning. The components were recognized by means of Wiley 2001 library data (NIST 02 version 2.62) of the GC-MS system, literature data, and comparison of the components’ Kovats indices (KIs) and mass spectra with those of standards. In order to calculate KI, alkanes (C5–C24) were employed as reference points. Every determination was made twice and then averaged.

### 2.10 Vitamins

Carotene and β-cryptoxanthin contents were obtained in acetone and examined by a reversed-phase HPLC conducted with a diode array detector (Sarungallo et al., 2015). To extract carotene and β-cryptoxanthin, seed samples were shaken in a MagNa Lyser (3 × 10 s, 6,000 rpm) using acetone as a solvent. After centrifugation, the sample was brought in the autosampler of the HPLC (Shimadzu SIL10-ADvp) and kept at 4°C. The separation of the carotenoids and β-cryptoxanthin was performed using a reversed-phase method with a low-pressure gradient and was performed on a silica-based C18 column (Waters Spherisorb 5-µm ODS1 4.6 × 250 mm). Solvent A (acetonitrile:methanol:water, 1:9:10) and solvent B (methanol:ethyl acetate, 68:32) act as the mobile phase. The detection of the carotene and β-cryptoxanthin was performed by a diode array detector (Shimadzu SPD-M10Avp) at a wavelength range of 446–470 nm and integrated *via* the software program (Shimadzu Lab Solutions Lite).

Phylloquinone was detected according to the methods of Jakob et al (Jakob and Elmadfa, 1996). A reversed-phase HPLC system and samples were separated on analytical column Gynkotek ODS Hyper-sil (250 · 4.6 mm i.d., 5 lm), a guard-column Gynkotek ODS Hypersil (20 · 4.6 mm i.d., 5 lm, Bischoff, Leonberg, Germany). The mobile phase contained 1 L of dichloromethane–methanol mixture (ratio 1:9). This solvent was then combined with 5 ml of a methanolic solution containing l.37 g of ZnCl_2_, 0.41 g of CH_3_COONa, and 0.30 g of CH_3_COOH. Recognition was performed at 243-nm excitation and 430-nm emission. The concentrations were calculated using a linear regression curve from standard solutions.

Tocopherols were obtained with hexane by means of the MagNa Lyser. The dried extract (CentriVap concentrator, Labconco, KS, USA) was resuspended in hexane, and tocopherols were isolated and estimated by HPLC (Shimadzu, ‘s Hertogenbosch, The Netherlands) (normal phase conditions, Particil Pac 5 µm column material, length 250 mm, i.d. 4.6 mm). Dimethyl tocol (DMT) was used as an internal standard (5 ppm). Data were investigated with Shimadzu Class VP 6.14 software.

### 2.11 UHPLC–MS/MS determination of phenolics and flavonoids

For quantification of individual phenolic acids and flavonoids, 0.3 g of dried powdered seeds or fruits was extracted in 2 ml of 80% (v/v) ethanol in a water bath at 70°C for 0.5 h. After centrifugation (12,000 rpm for 30 min), the supernatant was concentrated by a rotary evaporator (IKA-WERKE-RV06ML; Staufen, Germany). The obtained residue was dissolved in HPLC-grade methanol (final concentration = 1,000 ppm). All solutions were filtered through a 0.45-μm membrane filter (Iwaki Glass) before analysis. An Acquity UPLC System from Waters (Milford, CT, USA) was used for the extract’s chromatographic analysis. This system is supplied with a binary solvent supply system, degasser, autosampler, and column heater. Utilizing a 100 mm × 2.1 mm Acquity BEH C18 column from Waters with 1.7-m particle size, chromatographic separation was carried out. A Waters (Manchester, UK) Xevo TQD tandem quadrupole mass spectrometer was used for MS/MS detection together with an electrospray ionization interface (ESI) that worked in the negative ion mode. The capillary voltage was 4.5 kV, the source temperature was 120°C, the desolvation gas temperature was 400°C, and the nitrogen flow rates for the cone and desolvation gases were 30 and 600 L/h, respectively. Eluent A, ultrapure water containing 0.1% formic acid, and Eluent B, acetonitrile, were the components of the mobile phase. Samples measuring 2 μl were introduced at a flow rate of 0.2 ml/min with a linear gradient starting at 3% B and escalating to 100% B in 10 min. The presence of phenolic chemicals in the sample was confirmed using the multiple-reaction monitoring (MRM) mode, together with *m*/*z* transitions of the precursor ions and product ions.

3,5-Dichloro-4-hydroxybenzoic acid (Sigma-Aldrich Co., St. Louis, MO, USA) was utilized as an internal standard to take into consideration recovery losses and ionization efficiencies. The concentrations were calculated based on the relative response of the internal analyte in relation to this internal standard added. The amount of internal tracer is always chosen in relation to the internal concentration of the analyte, taking into account that the concentration difference does not exceed a factor of 100, the range in which linearity is guaranteed.

### 2.12 DPPH antioxidant assay

Seven samples (300 µl of methanolic extract) were mixed with 900 µl of DPPH solution (4 × 10^−5^ M). After incubation at 60 nm in the dark at 37°C, the absorbance was measured at 517 nm with a spectrophotometer UV–visible (A_(sample)_). A blank solution was also measured at the same wavelength (A_(blank)_). The free radical-scavenging activity of each solution was then calculated as percent inhibition according to the following equation: (radical scavenging activity (%) = ((A_(blank)_ − A_(sample)_)/A_(blank)_) × 100).

### 2.13 FRAP assay

The ferric ion-reducing antioxidant power (FRAP) activity was determined in the alcoholic (80% ethanol) seed and fruit extracts, which showed the highest activity by FRAP methods (AbdElgawad et al., 2019). FRAP assay was performed by adding 20 μl of each extract to a micro-titer plate and filling up to 200 μl of freshly prepared pre-warmed FRAP reagent. The mixture was incubated for 30 min at 37°C. The absorbance was determined at 593 nm. The antioxidant capability of the extracts was measured using the Trolox calibration curve.

### 2.14 TBARS lipid peroxidation assay

The level of lipid peroxidation was assessed by the thiobarbituric acid reactive substance (TBARS) method, using an egg yolk homogenate as the lipid-rich medium (Ohkawa et al., 1979). The seed extract and egg homogenate (0.5 ml of 10% v/v) were mixed with 15 mM of ferrous sulfate (to induce lipid peroxidation), and after 0.5 h, 1.5 ml of 10% trichloroacetic acid (TCA) was added. The mixture was then added to a 1.5-ml solution of 0.67% TBA and then boiled for 0.5 h. The chromogen obtained was measured at 535 nm.

### 2.15 Antimicrobial activity

The antibacterial activities of the seed and fruit ethanolic extracts were verified using the disc diffusion method (bacterial suspension containing 10^6^ CFU/ml of the bacterial test strain spread on Mueller–Hinton agar). Each extract was put on sterilized filter paper discs (5 μg/disc). Absolute ethanol was used as a negative control. These discs were placed on the agar plates to be incubated at 37°C for 24 h. The inhibition zones were measured by Vernier caliper. Regarding antifungal activity, a well diffusion assay was used to determine the antifungal properties of seed and fruit ethanolic extracts. Inoculum measuring 0.2 ml (fungal strain in saline) was spread on an agar plate. Five ditches of 4 mm were made on each plate. Absolute ethanolic plant extracts (50 mg/ml) were prepared, and each well was filled with 50 μl of the methanolic extracts. Fluconazole (5 mg/ml) was used as a positive control, while absolute ethanolic was used as a negative control. The plates were incubated at 37°C for 24 h. Strains of *Candida* species were obtained from the Research Laboratory, Clinical Laboratory Sciences Department, Juef University, Saudi Arabia.

### 2.16 *In silico* studies and molecular docking

The 3D structures of the proposed molecules were retrieved from the PubChem database and energy-minimized to investigate their interactions with microbial proteins through molecular docking analyses. The proposed molecules were five essential oils (cadinol, cryptone, elemol, thyme, and pinene), tocopherol (vitamin E), kaempferol (flavonoid), and glutamine (amino acid). Two microbial receptors were targeted: sterol 14-alpha demethylase from *Candida albicans* (Protein Data Bank (PDB): 5FSA (Hargrove et al., 2017)) and the transcriptional regulator MvfR of *P. aeruginosa* (PDB: 6Q7U (Zender et al., 2020)). The receptors were retrieved from the PDB database and prepared for docking through the removal of associated ligands and solvent molecules and the addition of polar hydrogen atoms. In AutoDock, the grid box was adjusted to target the entire protein target with a grid spacing of 1 Å between the set points (Morris et al., 2009). Docking simulations and binding affinity were performed and calculated using AutoDock Vina (Trott and Olson, 2010). The molecular interactions were further investigated by visualizing the formed chemical bonds in each ligand–receptor complex as well as the 3D surface structures showing the aromatic interactions, H-bond formation, ionizability, solvent-accessible surface (SAS), and other properties by using BIOVIA Discovery Studio (Gaber et al., 2020; Samaha et al., 2020; El Azab et al., 2021; Gaber et al., 2021; Mohamed et al., 2021; Saied et al., 2021; Healey et al., 2022; Khirallah et al., 2022a; Khirallah et al., 2022b).

### 2.17 Statistical investigation

Our tests were accomplished as quadruple. The data are shown as mean ± standard error of the mean. Results were investigated by using one-way ANOVA and subsequently Tukey’s *post hoc* test. The significance of the data was defined by *p*-value; *p* > 0.05 is regarded as non-significant and *p* < 0.05 as significant. The statistical investigation was performed using the software program GraphPad Prism (GraphPad Software, San Diego, CA, USA, 2007) (Abdel-Wahab BA et al., 2022; Mohamed et al., 2022b; Mohamed et al., 2022a).

## 3 Results and discussion

### 3.1 Proximate composition analysis

**Figure 1:**
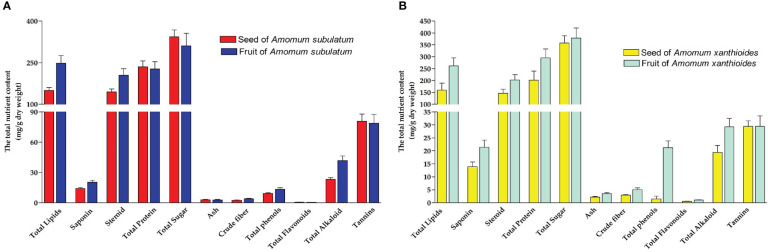
The total nutrient content (mg/g dry weight) in **(A)** seed and fruit of *Amomum subulatum* and **(B)** seed and fruit of *Amomum xanthioides*. The data are shown as the mean ± SEM of four plants per group.

For *A. subulatum*, sugar has the largest total content among other nutrients. For seeds, it was estimated to be approximately 33% of the total nutrient content in seeds, while that in fruits was approximately 25.5% of the nutrient content. Higher sugar content was found in *A. xanthioides* than *A. subulatum*. After sugars, both species contained considerable amounts of proteins and lipids. Notably, the protein content is usually higher than the total lipids in both species except the fruit of *A. subulatum*, which contains more lipids than proteins. However, lower amounts of steroids, tannins, and alkaloids were also observed. The investigation of alcoholic extracts of some *Amomum* species revealed the presence of various phytoconstituents, including flavonoids, carbohydrates, anthocyanin, tannins, phenols, alkaloids, and steroids (Konappa et al., 2020). Li et al. reported that *Amomum tsao-ko* is rich in a variety of chemical components of proteins, phenolic compounds, tannins, organic acids, saponins, flavonoids, anthraquinone, coumarin, lactones, steroids, terpenoids, volatile oil, anthocyanins, and so on (Li et al., 2021). As tannin, a wide group of water-soluble polyphenolics, and alkaloid possess anti-nutritional and anti-feed properties (Mueller-Harvey, 2006; Itkin et al., 2013), low availability of these compounds indicated high tissue quality of both *Amomum* species. However, tannins possessed antioxidant characteristics and have been shown to reduce total cholesterol, blood pressure, and immune system stimulation (Tong et al., 2021). Other nutrients are present in much lower concentrations including flavonoids, crude fiber, and ash (**Figures 1A, B**). The content of ash often indicated high levels of inorganic compounds and essential macroelements (Alzahrani et al., 2017).

This is consistent with the antioxidant activity associated with the ethyl acetate seed extract and the immune response activated in mice upon administration of fruit extracts (Sharma et al., 2017; Sudarsanan et al., 2021). Moreover, an anti-inflammatory function associated with tannins from various sources was reported in other studies (Park et al., 2014; Ambreen and Mirza, 2020; Sharma et al., 2021), which explains the beneficial use of *A. xanthioides* in the management of atopic dermatitis (Choi et al., 2017).

### 3.2 Essential oil isolation

Our data revealed that there are several essential oils found in both *A. subulatum* and *A. xanthioides* with different concentrations in both seeds and fruits (**Table 1**). Some of them showed significant differences between the two species in either seed or fruits, while the others showed no significant changes between the two species. High levels of thyme oil may promote the antimicrobial activity associated with *A. subulatum* and *A. xanthioides* (Satyal et al., 2012; Choi et al., 2017; Thinh et al., 2022). Thyme oil is used in food preservation and cosmetics due to its antibacterial, antifungal, and anti-inflammatory characteristics (Lorenzo et al., 2019). Thyme oil aids in promoting blood flow to the skin, which improves the healing process and removal of scars and imperfections, leaving the skin even and healthy (Zarzuelo and Crespo, 2002; Salehi et al., 2018). Reports of antibacterial activity of thyme oil have been found against *Clostridium botulinum*, a gram-positive bacterium that produces one of the most lethal neurotoxins known (De and De, 2019; Corsalini et al., 2021). In addition, thyme oil significantly reduced the growth of the fungus *Pyrenochaeta terrestris* during soil treatment, which suggests the potential use of thyme oil in the biological control of plant diseases (Hussien and Ibrahim, 2020). Due to their wide range of applications, *A. subulatum* and *A. xanthioides* can be used as sources for the extraction and production of thyme oil.

**Table 1:** Essential oil percentages (v/g dry weight) in the seed and fruit of *Amomum subulatum* and *Amomum xanthioides*.

Essential oil	*Amomum subulatum*		*Amomum xanthioides*	
	Seed	Fruit	Seed	Fruit
**γ-Terpinene**	5.63 ± 0.41	7.72 ± 0.85	7.46 ± 0.59	6.01 ± 0.66
**α-Thujene**	0.41 ± 0.04	0.54 ± 0.06	0.29 ± 0.09	0.45 ± 0.05
**α-Pinene**	17.01 ± 1.81	19.88 ± 2.58	12.82 ± 0.94	22.82 ± 2.9
**α-Phellandrene**	0.94 ± 0.09	1.44 ± 0.28	0.5 ± 0.04	1.59 ± 0.17^b^
**Myrcene**	4.29 ± 0.31	4.73 ± 0.74	3.17 ± 0.34	5.49 ± 0.78
**Sabinene**	1.07 ± 0.08	3.91 ± 0.88^a^	2.74 ± 0.29	3.38 ± 0.61
**β-Pinene**	4.54 ± 0.34	7.9 ± 0.96	2.3 ± 0.69	1.26 ± 0.49
**Carvacrol**	13.01 ± 1.63	11.08 ± 1.6	0.86 ± 0.08 ^a^	0.25 ± 0.03
***p*-Cymene**	5.34 ± 0.49	5.27 ± 0.61	3.48 ± 0.25	1.88 ± 0.05 ^b^
**β-Phellandrene**	0.16 ± 0.02	0.16 ± 0.02	0.14 ± 0.02	0.3 ± 0.04
**Thyme**	50.96 ± 5.91	78.75 ± 8.61	48.98 ± 5.67	48.65 ± 5.43
**Elemol**	18.82 ± 1.69	33.22 ± 2.99	18.96 ± 3.12	34.89 ± 6.26
**Myrtenal**	1.81 ± 0.17	2.12 ± 0.19	1.62 ± 0.12	2.21 ± 0.19
**Verbenone**	1.44 ± 0.15	1.64 ± 0.15	1.07 ± 0.08	1.87 ± 0.18
**Cuminal**	2.09 ± 0.16	2.36 ± 0.21	1.47 ± 0.14	2.72 ± 0.28
**Ascaridole**	0.43 ± 0.03	0.66 ± 0.11	0.47 ± 0.03	0.68 ± 0.09
***E*-nerolidol**	0.45 ± 0.03	0.91 ± 0.12	0.4 ± 0.08	0.36 ± 0.08
**Widdrol**	1.4 ± 0.15	1.46 ± 0.12	0.25 ± 0.05	0.12 ± 0.04
***epi*-Cubenol**	1.47 ± 0.13	1.25 ± 0.13	0.35 ± 0.03	0.18 ± 0.01 ^c^
**γ-Eudesmol**	0.44 ± 0.04	0.42 ± 0.03	0.29 ± 0.02	0.18 ± 0.01
***epi*-α-Muurolol**	4.09 ± 0.47	6.06 ± 0.43	3.93 ± 0.45	3.76 ± 0.28
**β-Eudesmol**	5.58 ± 0.6	8.7 ± 0.65	5.43 ± 0.41	6.41 ± 0.67
**α-Cadinol**	9.57 ± 0.91	16.31 ± 1.38	9.54 ± 1.17	15.26 ± 2.31
**Oplopanone**	0.55 ± 0.05	0.77 ± 0.06	0.52 ± 0.05	0.55 ± 0.04
**Pinocarvone**	0.26 ± 0.03	0.3 ± 0.03	0.22 ± 0.02	0.33 ± 0.03
**Cryptone**	15 ± 1.27	17 ± 1.52	10.78 ± 0.9	19.52 ± 1.43
**Linalool**	5.21 ± 0.37	2.96 ± 0.21 ^a^	5.29 ± 0.38	2.31 ± 0.17

The data are shown as the mean ± SEM of four plants per group. ^a^p < 0.05 versus seeds of A. subulatum. ^b^p < 0.05 versus seeds of A. xanthioides. ^c^p < 0.05 versus fruits of A. subulatum.

After thyme, elemol oil is the next-most prevalent oil in both species. The percent of elemol oil in seed was approximately 19%, while that in fruits was 34% for the two species. It is commonly used in fine fragrances as well as other applications (Bhatia et al., 2008). Elemol oil isolated from essential oils of *Rhynchanthus beesianus* rhizomes exhibited *insecticidal* activities against the adults of *Liposcelis entomophila* and *Tribolium castaneum* insects (Pan et al., 2022). Essential oils of *Cymbopogon schoenanthus* contained 22.8% elemol oil and exhibited larvicidal effects against *Anopheles funestus* and *Culex quinquefasciatus* larvae with lethal concentrations of 120.5 and 23.32 ppm, respectively (Wangrawa et al., 2022). In addition, elemol was the most abundant constituent (13.54%) in the essential oils of *Drimys winteri*, which showed insecticidal activity against the pests *Acanthoscelides obtectus* and *Aegorhinus superciliosus* (Tampe et al., 2020). The reported insecticidal activity of essential oils with elemol in multiple studies suggests its role as a natural eco-friendly insecticide for the biocontrol of herbivorous arthropods.

In addition to thyme and elemol components, considerable levels of the terpenoid α-pinene were also noticed in the two species. 1,8-Cineole, α-pinene, α-terpinene, and β-pinene were found to be the major components in *Amomum kravanh* (Diao et al., 2014). The oil has demonstrated a strong anticancer effect on various cell lines in addition to anti-inflammatory effects against multiple autoimmune disorders (Salehi et al., 2019; Prado-Audelo et al., 2021), which agrees with the high therapeutic activity of *A. subulatum*. Essential oils from *Magnolia candollei*, which contained 29.7% α-pinene and 10.2% elemol components, exhibited inhibitory against lipoxygenase and acetylcholinesterase, suggesting the potential therapeutic capabilities of these oils in diseases related to lipid metabolism as well as neurological disorders (Yahaya et al., 2022). Noticeably, the carvacrol levels in *A. subulatum* were 10 times higher than those in *A. xanthioides*. Lower levels of α-thujene, phellandrenes, ascaridole, and pinocarvone were also observed in both species. Other components were also isolated and summarized in **Table 1**. Statistical analyses showed that *A. subulatum* contained significant correlation levels (*p* < 0.05) of sabinene component in fruits more than in seeds while having linalool in seeds more than in fruits (*p* < 0.05). For *A. xanthioides*, significantly higher levels (*p* < 0.05) of α-phellandrene were observed in fruits. In addition, cymene seed levels were higher compared to those of fruits in *A. xanthioides*. When compared to *A. xanthioides*, *A. subulatum* showed higher significance (*p* < 0.05) of carvacrol in seeds and lower significance (*p* < 0.05) of cubenol in fruits. α-Pinene from some *Salvia* spp. showed moderate antibacterial activity against multiple bacterial species including *Bacillus subtilis*, *S. aureus*, and *Staphylococcus epidermidis* with inhibition zones ranging from 13 to 15 mm and minimum inhibitory concentration (MIC) values of 3.7–7.5 mg/ml (Yousefzadi et al., 2007). Moreover, α-pinene had a strong antifungal potential against *Candida* species isolated from otomycosis patients, indicating its promising clinical utility as a natural drug (Nóbrega et al., 2021). In this regard, it was suggested that essential oil might bind to the bacterial cell surface and then penetrate the phospholipid bilayer of the cytoplasmic membrane. Consequentially, it results in the leakage of various vital intracellular constituents and leads to cell death (Lv et al., 2011). Thus, the presence of α-pinene and elemol components in considerably high amounts in *A. subulatum* and *A. xanthioides* makes the plant species extremely important sources of these oils due to their great benefits in fighting both plant and human pathogens.

### 3.3 Elemental analysis

**Figure 2:**
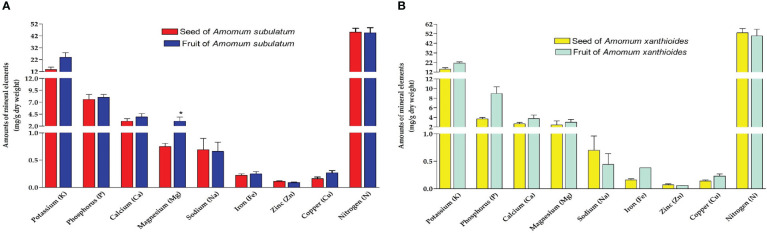
Amounts of mineral elements extracted (mg/g dry weight) in **(A)** seed and fruit of *A. subulatum*, **(B)** seed and fruit of *A. xanthioides*. The data are shown as the mean ± SEM of 4 plants per group. a *P* < 0.05 versus seeds of *A*. *subulatum*, c *P* < 0.05 versus fruits of *A. subulatum*.

In the seed and fruit of both species, nitrogen was the highest frequent element followed by potassium and then phosphorus (**Figures 2A, B**). *A. subulatum* contained lower levels of nitrogen (~45 mg/g dry weight) than *A. xanthioides* seed and fruit. Some elements were found in much lower amounts (less than 3 mg/g dry weight) including calcium, magnesium, sodium, and zinc. A significant increase in levels (*p* < 0.05) of magnesium was observed in the fruits of *A. subulatum* compared to seeds. However, zinc levels were significantly higher (*p* < 0.05) in fruits of *A. subulatum* compared to fruits of *A. xanthioides*. It was also reported that *Amomum longiligulare* is rich in microminerals (Chau et al., 2015). In this context, edible Zingiberaceae plants contained relatively high macroelement amounts including K, Ca, and Fe (Rachkeeree et al., 2018). Nitrogen is regarded as a critical macronutrient among all mineral nutrients including all living tissues within the plant, ranging from metabolism to resource allocation, growth, and development. Nitrogenous compounds play several roles in metabolism, including acting as protein building blocks and as precursors in the biosynthetic pathways of substances like lignin (Famiani et al., 2020; Yousaf et al., 2021). Phosphorus is a crucial nutrient in crop yields since many soils in their natural condition do not have enough accessible phosphorus to enhance crop yield (Sett and Soni, 2013). Potassium supplementation improved osmotic adjustment and water relations in a variety of crop species. K alleviates drought conditions in plant material primarily by managing stomatal closure and preserving stromal pH, thereby lowering photo-oxidative injury to chloroplasts (Santos et al., 2021). This means that these plants have excellent quality in improving the osmotic adjustment of water and preserving stromal pH.

### 3.4 Total phenolics and flavonoids

**Figure 3:**
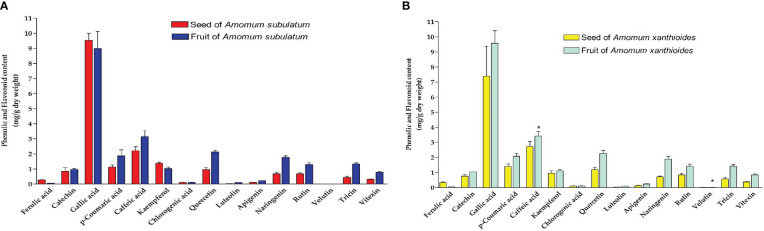
Phenolic and flavonoid content (mg/g dry weight) in **(A)** seed and fruit of *Amomum subulatum* and **(B)** seed and fruit of *Amomum xanthioides*. The data are shown as the mean ± SEM of four plants per group. ^b^ *p* < 0.05 versus seeds of *A. xanthioides*.

In determining the total phenolic and flavonoid components in both species *A. subulatum* and *A. xanthioides* in either seeds or fruits, we found that both species had no significant difference in the phenolic or flavonoid levels. However, there was a significant increase in the levels of caffeic acid and velutin (*p* < 0.05) in *A. xanthioides* fruits compared to seeds (**Figures 3A, B**). The phenolic–flavonoid profiling showed that only gallic acid, a phenolic compound, is produced in high amounts in the two species compared to the other screened compounds. Most of the phytochemical constituents in *Amomum* sp. are phenolics, flavonoids, and essential oils (Alkandahri et al., 2021). Smaller amounts of other phenolics including caffeic acid, ferulic acid, and catechin were found. Isolated flavonoids were quercetin, kaempferol, naringenin, luteolin, and apigenin. Similarly, quercetin was previously isolated from *A. longiligulare* (Chau et al., 2015).

Owing to its anticancer, antioxidant, antimicrobial, and anti-inflammatory capabilities, gallic acid has therapeutic applications in treating multiple medical conditions including GI infections, neurological diseases, and heart disorders (Kahkeshani et al., 2019; Shukla et al., 2022). Gallic acid was reported to induce apoptosis in leukemia HL60 cell lines owing to its antitumor and anti-proliferative effects observed in multiple studies (Maruszewska and Tarasiuk, 2019; Soto-Maldonado et al., 2019; Roba’ei et al., 2022). Gallic acid also showed potent anti-obesity properties by directly targeting adipose tissue and inhibiting lipogenesis (Dludla et al., 2018), which was reported upon administration of ethyl acetate extract of *A. xanthioides* to high-fat-diet mouse and rat models (Kim et al., 2015; Im et al., 2020). Regarding skin diseases, gallic acid was also reported to have an immunosuppressive role in the regulation of atopic dermatitis by inhibiting the production of certain pro-inflammatory cytokines and chemokines (Tsang et al., 2016). These studies further increase the medicinal importance of *A. subulatum* and *A. xanthioides* plants as sources of natural product-based drugs since they contain considerable amounts of gallic acid.

### 3.5 Vitamins

**Figure 4:**
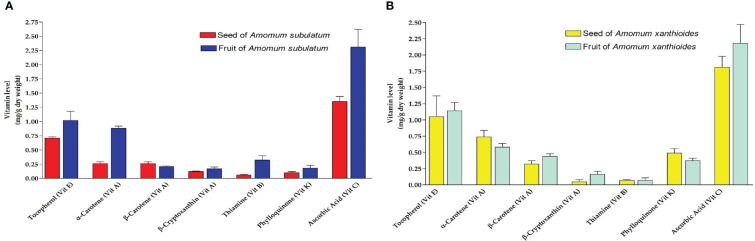
Vitamins levels (mg/g dry weight) isolated in **(A)** seed and fruit of *Amomum subulatum* and **(B)** seed and fruit of *Amomum xanthioides*. The data are shown as the mean ± SEM of four plants per group. ^a^ *p* < 0.05 versus seeds of *A*. *subulatum*, ^c^ *p* < 0.05 versus fruits of *A*. *subulatum*.

While screening for vitamins, small amounts of vitamins A, B, and K were found, which did not exceed 1 mg/g dry weight, while slightly higher amounts of vitamins C and E were found in seed and fruit of both species (**Figures 4A, B**). Despite the overall small amounts of vitamins, seeds of *A. xanthioides* had significantly (*p* < 0.05) higher levels of vitamin E and vitamin C in seeds when compared to *A. subulatum*. A significant increase (*p* < 0.05) of vitamin E and vitamin K levels was also observed in *A. xanthioides* fruits than in *A. subulatum* fruits. Vitamin E content in plants is thought to be linked to ripening and senescence. The non-volatile substances isolated from this *Amomum* including vitamins were also observed (Chau et al., 2015). Plants are subjected to oxidative stress throughout senescence, which leads to a rise in fatty acid-free radicals. Enhancing the content of vitamin E during senescence may be a stress-reduction technique for plants. In addition, by collaborating with other antioxidants, vitamin E can effectively eliminate the generation of reactive oxygen species (ROS). In plants, vitamin C is the most abundant and widely distributed water-soluble cellular antioxidant. Plants can avoid oxidative damage by directly scavenging ROS with vitamin C. Furthermore, vitamin C is essential for plant growth, development, and stress responses. It functions as a cofactor for many enzymes, regulates cell division, and influences cell expansion. It also regulates plant senescence (Paciolla et al., 2019; Zhang et al., 2020). These results revealed the antioxidant effects of these plants in the DPPH, FRAP, and anti-lipid peroxidation tests.

### 3.6 Amino acids

**Figure 5:**
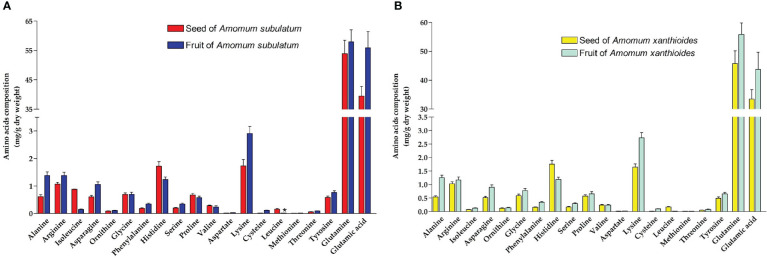
The amino acid compositions (mg/g dry weight) in **(A)** seed and fruit of *Amomum subulatum* and **(B)** seed and fruit of *Amomum xanthioides*. The data are shown as the mean ± SEM of four plants per group. ^a^ *p* < 0.05 versus seeds of *A*. *subulatum*.

Amino acid profiling revealed that both species possess small amounts of all amino acids except for glutamine and glutamic acid, which were found in much higher amounts in both seed and fruit (**Figures 5A, B**). Generally, *A. subulatum* had higher amounts of the two amino acids than *A. xanthioides*. Leucine levels were significantly increased (*p* < 0.05) in seeds of *A. subulatum* compared with the fruits of the same species. A comprehensive chemical investigation found that *A. tsao-ko* contained a variety of chemical components including protein and amino acids (Xie et al., 2022). Among the amino acids, glutamine serves as a major amino donor for the synthesis of amino acids, nucleotides, and other nitrogen-containing compounds, in addition to its role in nutrition and metabolism (Kan et al., 2015).

### 3.7 Antioxidant biological activity

Multiple tests revealed the antioxidant activity of seed and fruit extracts of both species (**Table 2**). According to the DPPH assay, the seed extract of *A. subulatum* exhibited the highest antioxidant activity followed by the seed extract of *A. xanthioides*. Similarly, FRAP assay showed that the seed extract of the two species had the best antioxidant activity, i.e., 20.14 ± 1.11 and 21.18 ± 1.04 µmol Trolox g^−1^ DW for *A. subulatum* and *A. xanthioides*, respectively. In terms of anti-lipid peroxidation, higher values were obtained from the fruit extract of *A. subulatum* and the seed extract of *A. xanthioides*. No statistical significance was found between the antioxidant activity of seeds and fruits in the same plant or the two species. These results indicate the high antioxidant activity of these extracts that contributes to their usefulness in a variety of therapeutic applications. It was also observed that plants of the genus *Amomum* showed antioxidant and anti-inflammatory activities (Kwon et al., 2003). The presence of gallic acid and tannins may be the main contributor to the high antioxidant activity associated with *A. subulatum*, which was previously reported in various studies (Bhaswant et al., 2015; Gautam et al., 2016; Sharma et al., 2017). The intermediate antioxidant activity of essential oils isolated from two *A. subulatum* fruit samples collected from India and Saudi Arabia was formerly reported (Alam and Singh, 2021). The free radical scavenging activity at 1,000 μg/ml was approximately 85.27% in the Indian sample and 86.86% in the Saudi Arabian sample, and the IC_50_ values were 219.38 and 203.79 μg/ml for the Indian and Saudi Arabian samples, respectively. For *A. xanthioides*, antioxidant activity was previously evaluated for the water and ethanol extracts using DPPH assay, which showed that the water extract had higher antioxidant activity than the ethanolic one with IC_50_ values of 2.87 and 3.31 µg/ml for water and ethanol extracts, respectively (Myint et al., 2012).

**Table 2:** Antioxidant activity exhibited by *Amomum subulatum* and *Amomum xanthioides* extracts.

Assay	*Amomum subulatum*		*Amomum xanthioides*	
	Seed	Fruit	Seed	Fruit
**DPPH (% inhibition)**	78.26 ± 9.27	63.27 ± 3.33	68.21 ± 2.56	51.53 ± 1.72
**FRAP (µmol Trolox g^−1^ DW)**	20.14 ± 1.11	13.19 ± 1.15	21.18 ± 1.04	15.66 ± 1.61
**Anti-lipid peroxidation**	4.87 ± 0.34	6.08 ± 0.35	6.11 ± 0.55	5.12 ± 0.8

The data are shown as the mean ± SEM of four plants per group.

FRAP, ferric ion-reducing antioxidant power.

### 3.8 Antimicrobial activity

**Figure 6:**
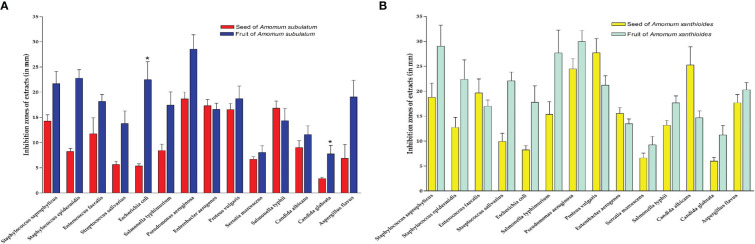
Inhibition zones (mm) of extracts in **(A)** seed and fruit of *Amomum subulatum* and **(B)** seed and fruit of *Amomum xanthioides*. The data are shown as the mean ± SEM of four plants per group. ^a^ *p* < 0.05 versus seeds of *A*. *subulatum.*.

Collectively, extracts from both species demonstrated strong antimicrobial effects against various pathogenic bacteria and fungi (**Figures 6A, B**). Ethanolic seed extracts of *A. subulatum* had the highest efficiency against four gram-negative bacterial species that cause serious human diseases, namely, *Enterobacter aerogenes*, *Proteus vulgaris*, *P. aeruginosa*, and *Salmonella typhimurium*. In addition, *P. aeruginosa* was also inhibited by the fruit extract of both *A. subulatum* and *A. xanthioides*. For the seed extract of *A. xanthioides*, large inhibition zones were formed against *P. vulgaris* and the fungus *C. albicans*. In this regard, *Amomum compactum* extracts damaged the membrane of fungal cells and inhibited the enzyme system of fungi to create a bland zone around the disc (Oh et al., 1951). The fruit extract of *A. xanthioides* caused high inhibition of *Staphylococcus saprophyticus* and *S. typhimurium* in addition to *P. aeruginosa*. While there were non-significant correlations between the antimicrobial activity of seeds and fruits between the two species, a statistically significant increase (*p* < 0.05) was observed in the antimicrobial activity of *A. subulatum* fruits with *E. coli* and *Candida glabrata* compared with seeds.

Indeed, the antimicrobial potential of both species was reported in various studies against the tested pathogens including *S. aureus* and *A. niger* (Satyal et al., 2012; Thinh et al., 2022). A concentration-dependent antibacterial activity was observed upon testing the essential oils of Indian and Saudi Arabian *A. subulatum* fruit samples against *Acinetobacter baumannii*, *E. coli*, and *P. aeruginosa*, with an inhibition zone range of 12–16 mm (Alam and Singh, 2021). In an older study, essential oils from Indian *A. subulatum* fruits exhibited antimicrobial activity similar to or higher than that of ciprofloxacin (as a standard) against *B. pumilus*, *S. aureus*, *S. epidermidis*, and *P. aeruginosa* (Agnihotri et al., 2012). For the seeds, methanolic seed extracts of *A. subulatum* exhibited reasonable antimicrobial activity at a dose of 800 mg/ml against *B. subtilis*, *Salmonella typhii*, and *P. aeruginosa*, when compared to tetracycline as a standard (Tijjani et al., 2012). Bacterial cell death might come from the effect of essential oil on membrane permeability, resulting in bacterial cell wall lysis (Bajpai et al., 2013). Generally, essential oils are considered the plant immune system due to their antimicrobial effect against a wide range of pathogens (Monica and Ioan, 2018). The pure form of essential oils from *A. subulatum* exhibited antimicrobial activity against various bacterial pathogens including *E. faecalis*, *S. aureus*, and *Shigella dysenteriae* forming inhibition zones of 14–23 mm (Tandukar et al., 2018). For the two species, the ratio of thyme oil was the highest among other oils in seed and fruit. In general, thyme oil and gallic acid could be the main factors promoting the antimicrobial properties of the two species due to their known antibacterial and antifungal activities and their high abundance among other oils (Borges et al., 2013; Sarjit et al., 2015; Lorenzo et al., 2019).

### 3.9 Molecular docking simulations

**Figure 7:**
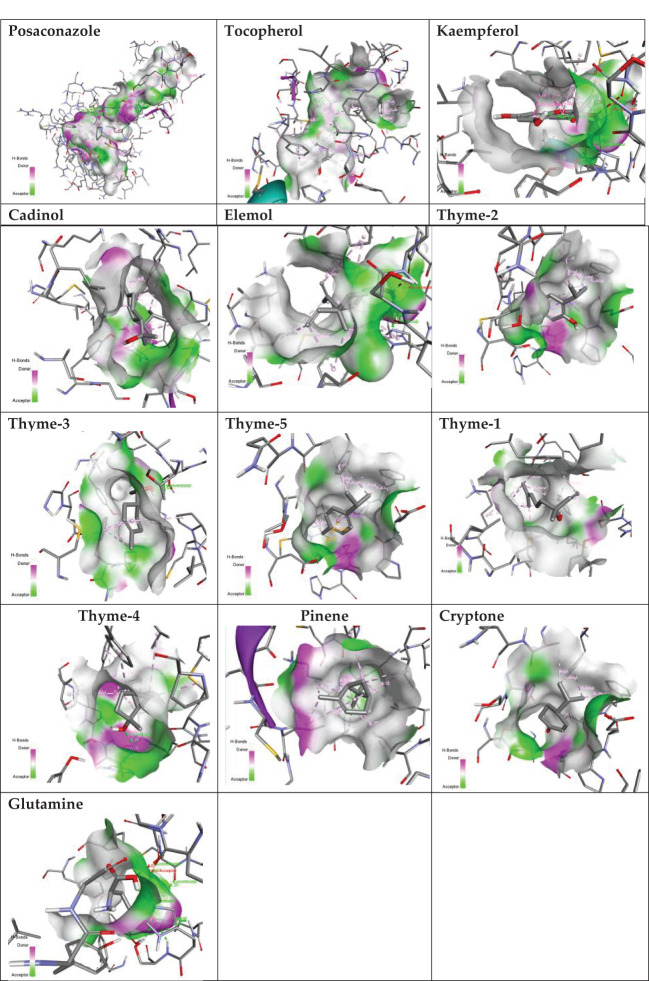
Hydrogen bond donor/acceptor surface view of the proposed molecules on sterol 14-alpha demethylase.

**Figure 8:**
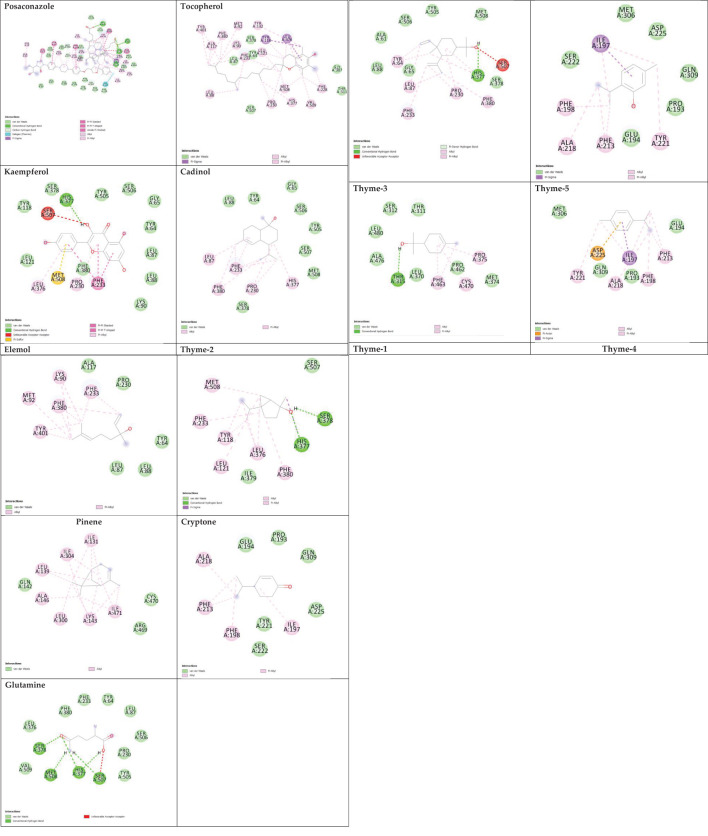
2D ligand interaction diagram of the proposed molecules with sterol 14-alpha demethylase.

Sterol 14-alpha demethylase of the fungus *C. albicans* is a common target of azole drugs since it is essential in maintaining the fungus cell membrane integrity through catalyzing a vital step in ergosterol synthesis (Hargrove et al., 2017; Marek and Timmons, 2019; Zhang et al., 2019). The docking results of the proposed molecules are summarized in **Table 3**. The standard antifungal drug, posaconazole, formed the highest interaction with the receptor, which was indicated by the low binding affinity (−15.2 kcal/mol), followed by tocopherol (−9.6 kcal/mol) and the flavonoid kaempferol (−8.4 kcal/mol). The hydrophobicity of 3D surfaces shows that more hydrophobic interactions were formed between the ligands and the target molecules (**Figure 7**). Aromatic interactions visualize the most stable conformation of each ligand in the binding pocket with respect to the whole protein molecule (**Figure S2**). SAS structures show that a large surface area, shown in blue, is involved in the ligand binding, which increases the binding efficiency and further stabilizes the ligand in the protein binding site (**Figure S3**). Since the interactions mainly depended on the hydrophobic interactions, no formal charges or ionized bonds were formed, and a relatively neutral interface (white) is formed between the ligands and the receptor (**Figure S4**). Some molecules, including kaempferol, elemol, and glutamine, formed polar hydrogen bonds with the target receptor, which decreased the binding energy and contributed markedly to stabilizing the formed complex. The hydrogen bond donor/acceptor surface showed the polar areas (pink/green) between the ligand and the receptor due to the formation of hydrogen bonds (**Figure S5**). The 2D interaction diagram (**Figure 8**) showed the specific positions of amino acids involved in the interaction with each molecule as well as the types of chemical bonds formed between them in addition to their distances stated in **Table 4**.

**Table 3:** Docking results of the proposed molecules with transcriptional regulator MvfR (PDB: 6Q7U).

Name	Binding affinity	Hydrophobic residues	Distance	H-bond residues	Distance
**HQQ**	−8	LEU197- LEU208- ALA168- PRO238- ILE236- PRO129- ALA130	3.96076- 3.88127- 4.29327- 4.05885- 5.32035- 5.00021- 4.83028	–	–
**Tocopherol**	−8.3	LEU207- ALA102- ALA130- ALA168- ILE263- ILE149- PRO238- LEU197- LEU208- ILE236- PHE221- TYR258-	3.78142- 3.90905- 3.68213- 4.23219- 5.19753- 4.5559- 3.75019- 3.93976- 3.77058- 4.71286- 4.83515- 5.26525	–	–
**Kaempferol**	−8.2	ILE236- ALA168- LEU208- ALA102- ILE149- PRO238	3.55105- 5.47727- 5.29956- 4.45556- 4.3678- 4.76949	LEU207	2.58672
**Cadinol**	−7.7	ALA102- ALA168- LEU208- ILE236- ILE149- LEU197- PRO129- LEU208 PHE221	4.40483 4.41751 5.2447 4.02302 4.90871 4.30594 3.89275 3.92425 5.33667	–	–
**Thyme-2**	−6.8	LEU208 PHE221 ALA130 ALA168 PRO129 LEU197 ILE149 ILE236 PRO238 PRO129 LEU197	3.72919 5.10225 3.79191 4.29797 4.45527 3.69787 5.37132 4.76293 4.11078 4.9306 4.83712	–	–
**Cryptone**	−6.7	LEU197 LEU208 ILE236	4.605 4.22898 3.71001	ALA130	2.32825
**Thyme-5**	−6.7	LEU208 PHE221 ALA130 ALA168 LEU197 LEU208 ILE149 ILE236 PRO238 PRO129	3.65717 5.2505 3.81477 4.28436 3.82674 3.83104 5.27522 4.86006 4.17347 5.03709	–	–
**Thyme-3**	−6.6	ALA130 LEU197 LEU208 PRO129	3.77576 4.11458 4.22878 3.43155	GLN194 SER196 LEU208	2.95299 2.66477 2.66181
**Elemol**	−6.3	ALA102 ALA168 LEU208 ILE236 ILE149 PRO238 PRO129 LEU207 PHE221	4.26534 4.49849 4.91296 4.30588 5.14165 4.3817 4.54934 4.0581 4.85289	–	–
**Thyme-4**	−6.1	ALA130 LEU197 LEU208 PRO129 ILE149 PHE221	4.35225 5.41336 4.11982 3.64299 5.2382 5.4972	ILE236	2.42809
**Pinene**	−6	LEU208 ILE236 MET224 LEU197 PRO129 PHE221	5.01287 4.72332 5.08638 4.39848 4.14459 4.90766	**-**	**-**
**Thyme-1**	−5.9	ALA130 ALA168 ILE149 LEU197 LEU208 PRO129	3.71024 3.61391 4.78145 4.39862 4.07807 3.80864	**-**	**-**
**Glutamine**	−5.5	–	–	GLN194 LEU197:O LEU208:O HIS204:ND1 SER196	2.6024 2.85136 2.82554 2.73285 3.75025

**Table 4:** Docking results of the proposed molecules with sterol 14-alpha demethylase (PDB: *5FSA*).

Name	Binding affinity	Hydrophobic residues	Distance	H-bond residues	Distance
**Posaconazole**	−15.2	THR311 TYR64 PHE233 ALA61 PHE463 ARG469 ALA62 PRO230 ALA476 LEU370 PRO375 LEU376 ILE379 ILE471 ILE304 PHE58 TYR118 TYR132 HIS377 CYS470 ILE131 LEU121 MET508	3.90404 5.7893 5.48366 4.8006 4.63529 4.8041 4.46946 5.15083 4.37419 5.2972 3.72448 4.6252 4.56791 4.27882 4.67822 4.82018 4.67667 5.46517 4.45682 3.95546 5.2038 5.32755 5.47494	TYR132 ARG381 HIS468 TYR118 GLY307 MET508 HIS377	2.64558 2.24954 2.16623 3.06271 3.60646 3.74934 3.17384
**Tocopherol**	−9.6	LEU376 TYR118 ALA117 LEU121 LEU376 PRO230 MET508 VAL509 LEU88 LYS90 MET92 TYR118 TYR132 PHE228 PHE233 HIS377 PHE380 TYR401	3.73737 3.82059 3.73786 4.95867 4.96483 4.70491 3.96657 4.80687 5.13365 4.65839 4.70436 5.24085 5.43382 4.89369 4.86514 4.49524 4.66532 4.53285	–	–
**Kaempferol**	−8.4	PHE233 - PRO230 LEU376	4.76319 5.07271 5.07682	HIS377	2.41623
**Cadinol**	−7.5	LEU87 PRO230 PHE233 HIS377 PHE380	5.39462 5.02188 4.01347 4.60132 4.41745	**-**	**-**
**Elemol**	−7.5	PRO230 LEU87 TYR64 PHE233 HIS377 PHE380	5.18674 5.00227 4.19177 4.37583 4.96068 4.88153	HIS377	2.54609- 2.96656
**Thyme-2**	−6.4	ILE197 ALA218 PHE198 PHE213 TYR221	3.94221 4.3587 4.3256 4.57551 4.9718	–	–
**Thyme-3**	−6.4	PRO375 CYS470 PHE463	4.86757 5.10794 5.4915	THR315	2.31527
**Thyme-5**	−6.1	ILE197 ALA218 PHE198 PHE213 TYR221	3.94487 4.46078 4.307 4.57848 4.93474	**-**	**-**
**Thyme-1**	−5.9	LYS90 MET92 PHE233 PHE380 TYR401	4.62746 4.7503 4.40604 4.92622 4.26215	–	**-**
**Thyme-4**	−5.9	HIS377 LEU121 LEU376 MET508 LEU376 TYR118 PHE233 PHE380	3.86265 5.46847 5.06807 4.44248 4.01994 4.56292 4.82083 5.13759	HIS377 SER378	3.06152 2.23329
**Pinene**	−5.8	ILE131 LYS143 ALA146 ILE304 ILE471 LEU139 LEU300	5.10348 5.19261 4.32492 5.29182 5.25868 4.98531 5.15443	**-**	**-**
**Cryptone**	−5.7	ALA218 PHE198 ILE197 PHE213	3.95208 5.06528 4.62198 4.85444	**-**	**-**
**Glutamine**	−5.2	–	–	HIS377 SER378 SER507 MET508	3.03508 2.29239 2.61005 2.25831

**Figure 9:**
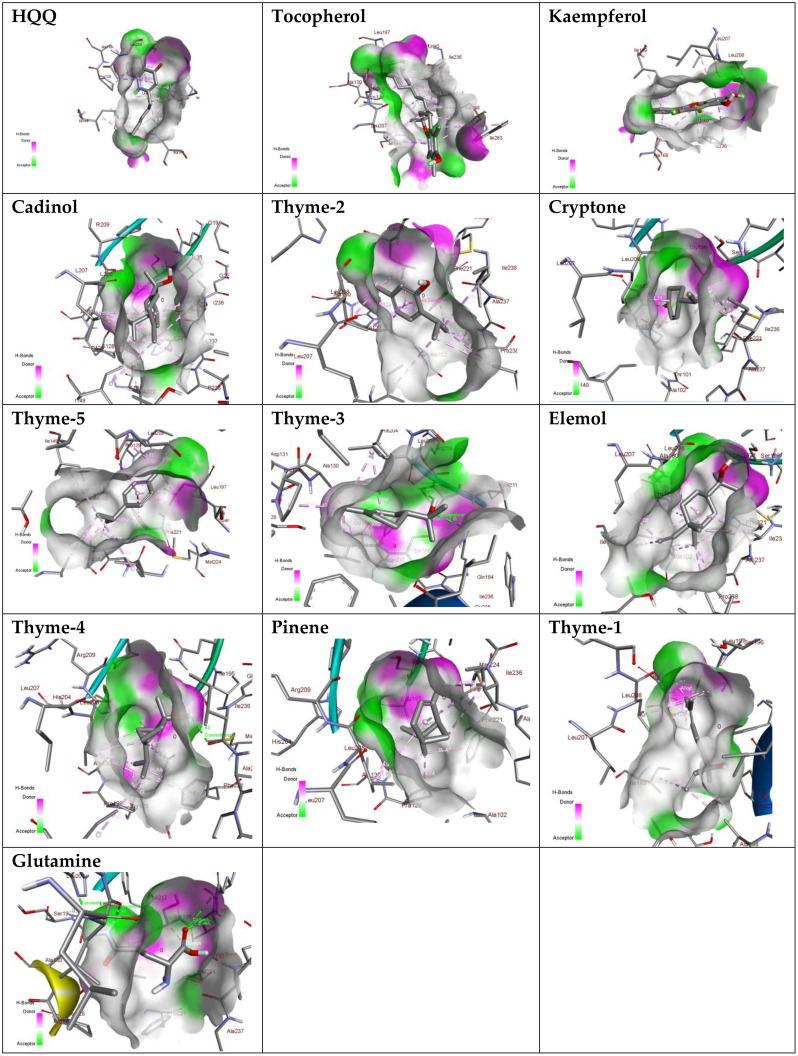
Hydrogen bond donor/acceptor surface view of the proposed molecules on MvfR.

**Figure 10:**
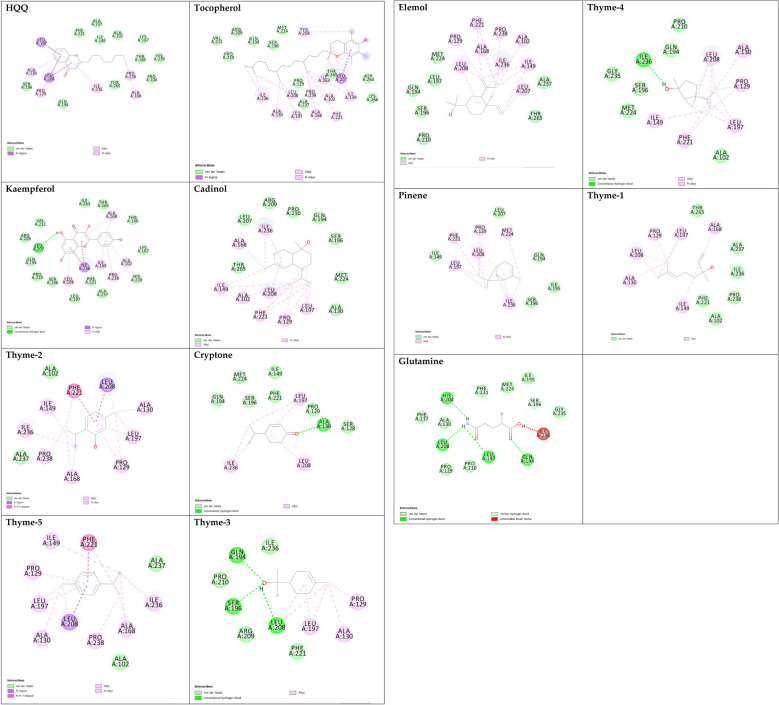
2D ligands interaction diagram of the proposed molecules with MvfR.

The transcriptional regulator MvfR of *P. aeruginosa* is responsible for the regulation of multiple virulence genes that are responsible for the pathogenicity of *P. aeruginosa*; therefore, it is an important drug target (Déziel et al., 2005; Allegretta et al., 2017; Zender et al., 2020). Similar to the demethylase, the interaction of the proposed molecules with the MvfR was dependent on hydrophobic interactions rather than hydrophilic ones, except for glutamine. The docking results, in **Table 3**, showed that tocopherol and kaempferol formed stronger and more stable complexes with the receptor than the antibacterial agent 2-heptylquinolin-4(1*H*)-one (HQQ), which indicates the high potency of these compounds and their promising antibacterial capabilities. The binding affinity was −8.3 and −8.2 kcal/mol for tocopherol and kaempferol, respectively, while for HQQ, it was −8 kcal/mol. In addition, more residues were involved in the hydrophobic interactions between tocopherol and MvfR, while the kaempferol–MvfR complex involved the formation of one hydrogen bond in addition to the hydrophobic one, which further stabilizes the ligand through partial charge formation. A large hydrophobic surface was observed between the ligands and the receptor (**Figure S6**). Aromatic surfaces show the conformation of the docked molecules responsible for the calculated binding affinity (**Figure S7**), and the large solvent-accessible area further confirms the stable complex (**Figure S8**). As expected, very low charge bonds are formed between the ligands and the receptors since the reactions relied mostly on hydrophobic interactions leading to the formation of neutral interfaces (**Figure S9**). The hydrogen bond formation was observed in the cases of kaempferol, cryptone, thyme-3,4, and glutamine, which are shown in the hydrogen bond donor/acceptor surface view in **Figure S10**. The stable ligand conformation was increased due to the formation of multiple chemical bonds with many atoms of the ligand that hold the ligand steady and make it more stable in the binding site. This was highly observed in tocopherol, cadinol, and elemol (**Figures 9**, **10**).

## 4 Conclusion

This study showed the composition and the major constituents of the medicinal plants *A. subulatum* and *A. xanthioides* as well as the therapeutic functions associated with them. Both species are considered good sources of antimicrobials including gallic acid, α-pinene, and thyme oil. The most effective antibacterial agents were ethanolic seed extracts of *A. subulatum* against *E. aerogenes*, *P. vulgaris*, *P. aeruginosa*, and *S. typhimurium*, while the antifungal effect was demonstrated by α-pinene oil against *Candida* species. The strong antioxidant activity was associated with tannins and gallic acid. Gallic acid had various medicinal benefits including apoptotic and anti-proliferative effects on cancer cells, potent anti-obesity activity on fatty mice and rats, and an immunosuppressive role in the regulation of skin diseases. The anti-inflammatory potential associated with essential oils, namely, thyme and α-pinene oils, can also be employed in immunosuppressive drugs. Finally, detailed molecular modeling studies indicated that the antimicrobial activities of these plants could be attributed to the high binding affinity of their bioactive compounds to bind to the active sites of the sterol 14-alpha demethylase and the transcriptional regulator MvfR. These findings further emphasize the significance of *A. subulatum* and *A. xanthioides* plants for the formulation and production of novel, less toxic, and more efficient pharmaceuticals in the future. However, clinical and *in vivo* studies are needed to validate the proposed therapeutic applications.

## Data availability statement

The original contributions presented in the study are included in the article/**Supplementary Material**. Further inquiries can be directed to the corresponding authors.

## Author contributions

All authors contributed to the article and approved the submitted version.

## In memoriam

This article is dedicated to the memory of Prof. Fatimah O. Nassief, former Vice president of female campus at KAU and co-founder of the Prophetic Medicine Chair for her dedicated life in serving science and humanity.
