# Supplementary material for: Insight into the phytochemical profile and antimicrobial activities of Amomum subulatum and Amomum xanthioides: an in vitro and in silico study
Source: Front Plant Sci. 2023 Apr 20;14:1136961. doi: 10.3389/fpls.2023.1136961 (PMC10157186; doi:10.3389/fpls.2023.1136961)
Supplement: Supplementary file 1 [file DataSheet_1.pdf]

Supporting Information

Insight Phytochemical Profile and Antimicrobial Activities of *Amomum subulatum* and *Amomum xanthioides; in vitro* and *in silico* study

Mohammed H. Alruhaili[1,2,*], Mohammed S. Almuhayawi[1], Hattan S. Gattan[2,3], Mohanned Talal Alharbi[4], Mohammed K. Nagshabandi[4], Soad K. Al Jaouni[5], Samy Selim[6,*], Hamada AbdElgawad[7*]

**Posaconazole**	**Tocopherol**	**Kaempferol**
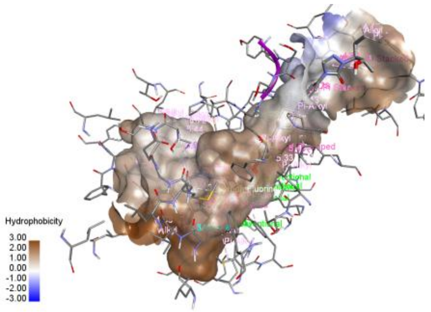	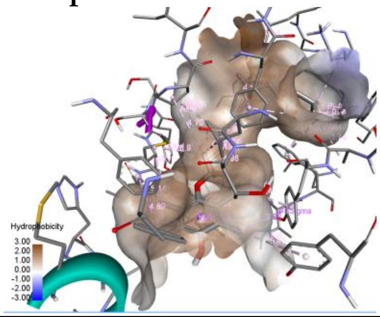	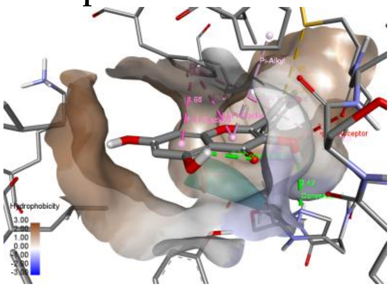
**Cadinol**	**Elemol**	**Thyme-2**
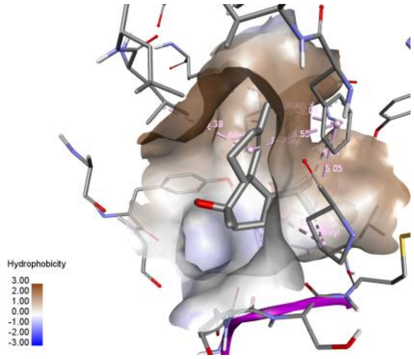	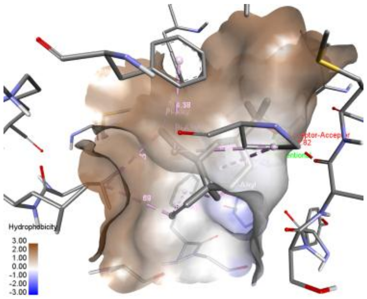	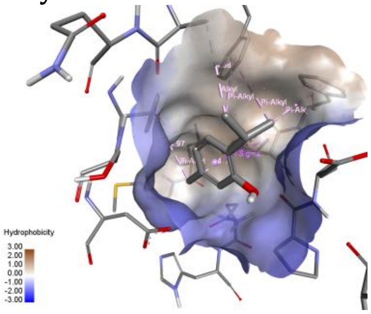
**Thyme-3**	**Thyme-5**	**Thyme-1**
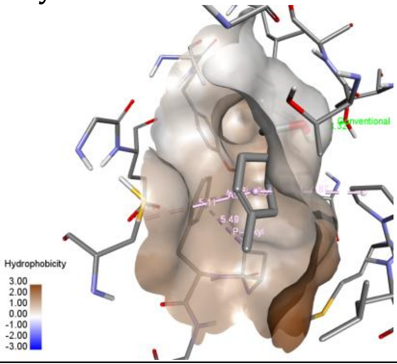	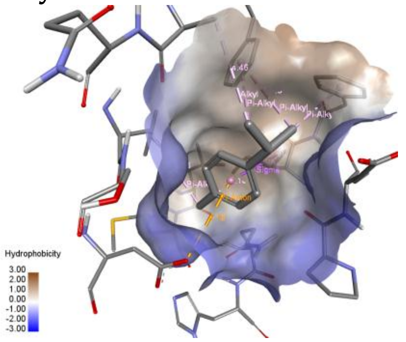	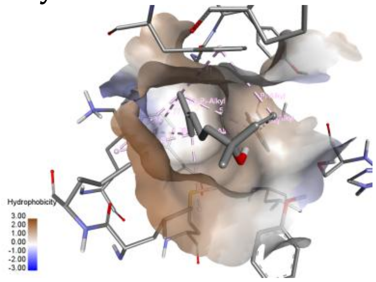

Thyme-4

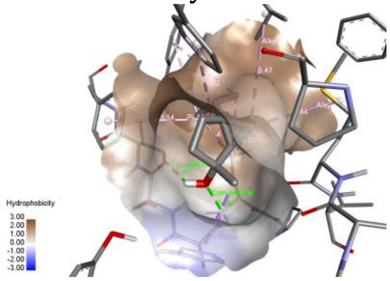

Pinene

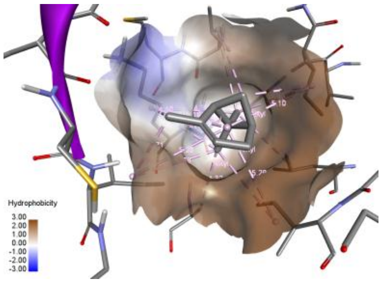

Cryptone

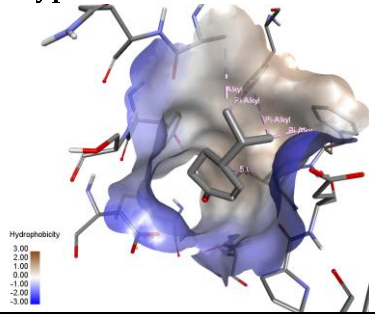

Glutamine

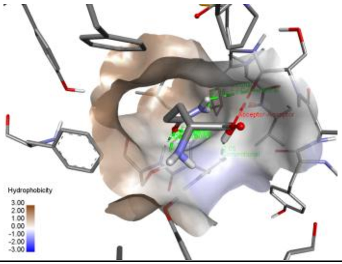

Figure S1: Hydrophobic interfaces involved in the interaction of the proposed molecules with Sterol 14-alpha demethylase.

Posaconazole

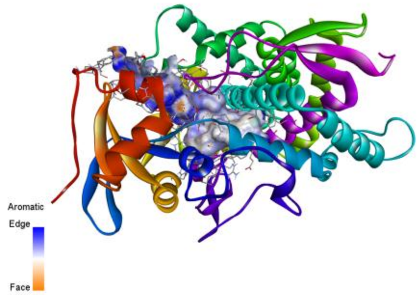

Tocopherol

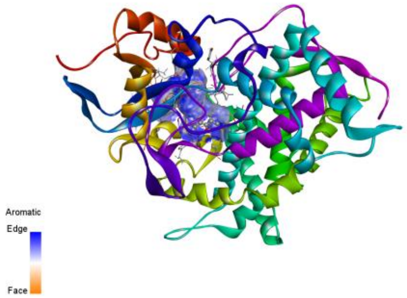

Kaempferol

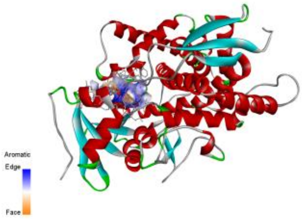

Cadinol

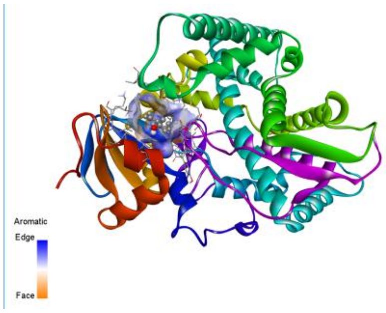

Elemol

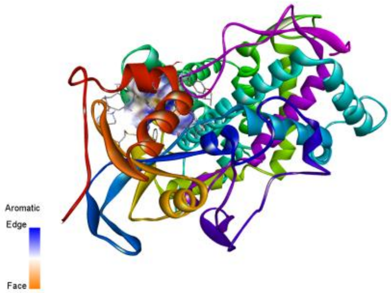

Thyme-2

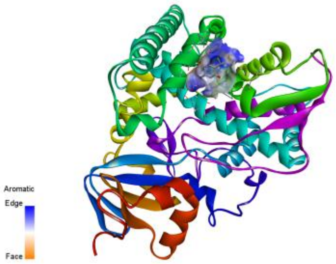

Thyme-3

Thyme-5

Thyme-1

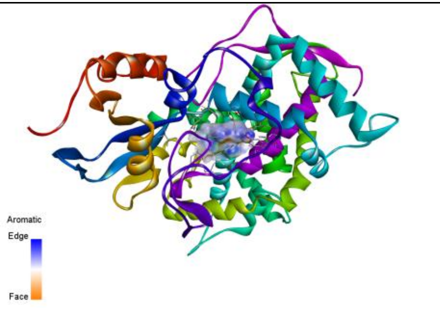	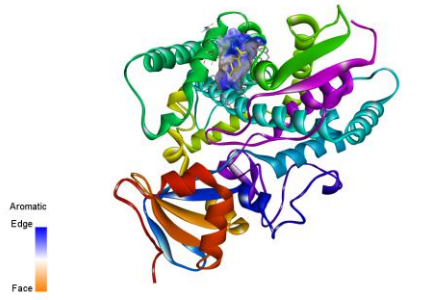	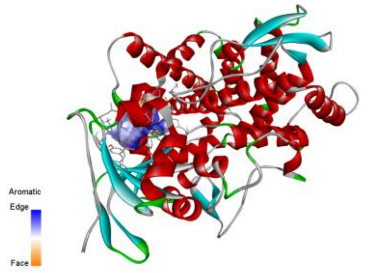
**Thyme-4** 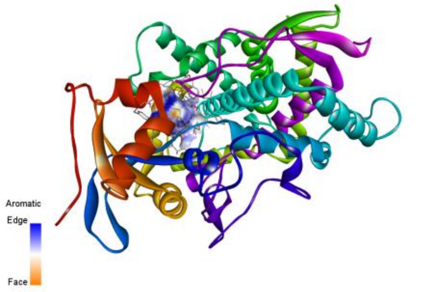	**Pinene** 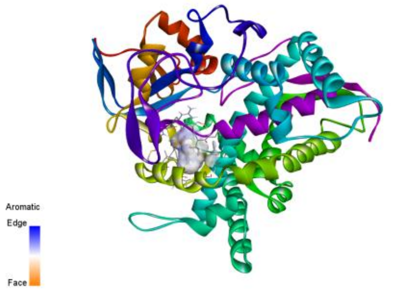	**Cryptone** 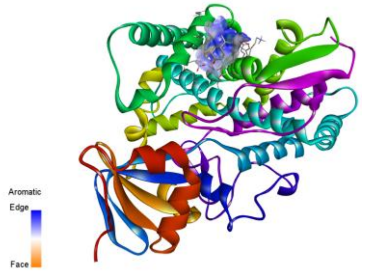
**Glutamine** 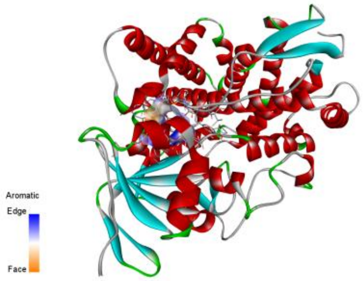		

Figure S2: Aromatic surface view of the proposed molecules on Sterol 14-alpha demethylase.

**Posaconazole** 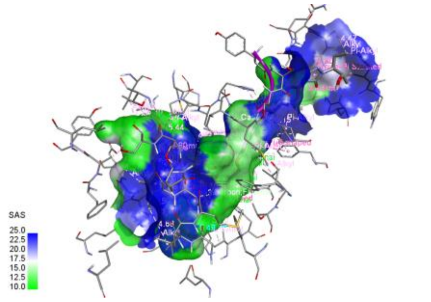	**Tocopherol** 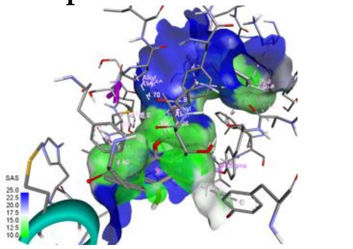	**Kaempferol** 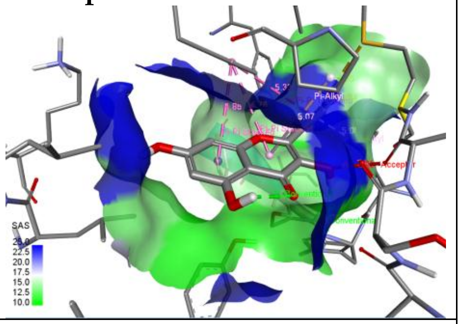
**Cadinol** 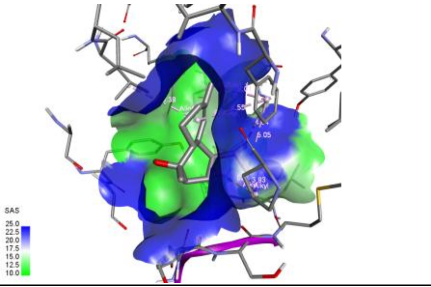	**Elemol** 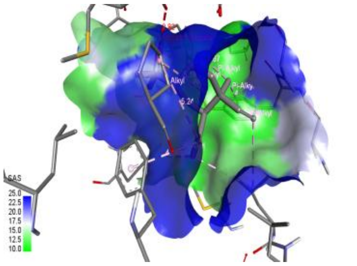	**Thyme-2** 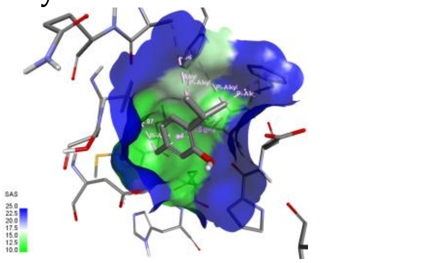
**Thyme-3**	**Thyme-5**	**Thyme-1**

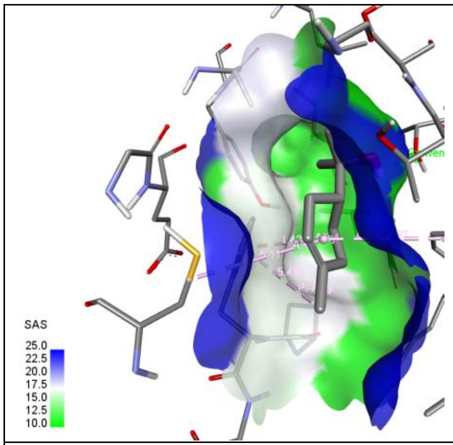	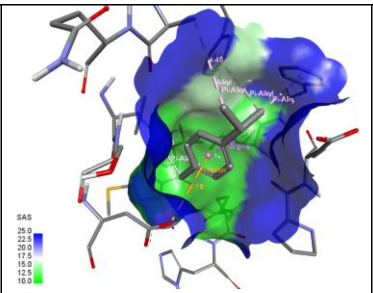	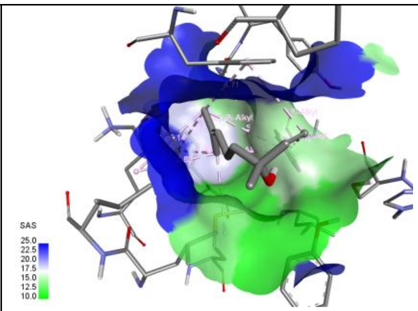
**Thyme-4** 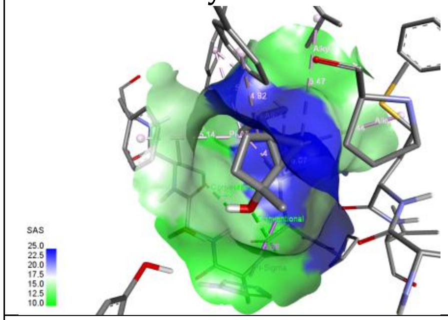	**Pinene** 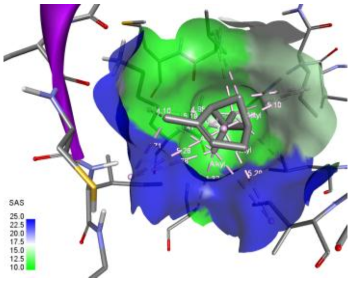	**Cryptone** 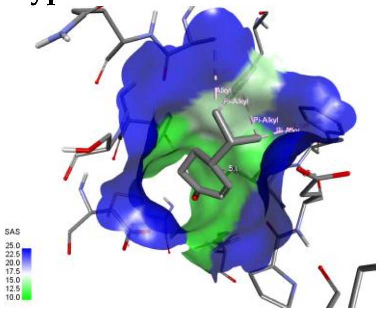
**Glutamine** 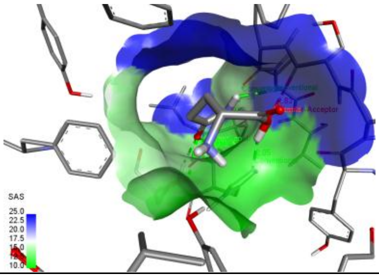		
**Figure S3:** Solvent-accessible area on the interface between of the ligands and Sterol 14-alpha demethylase.		

**Posaconazole** 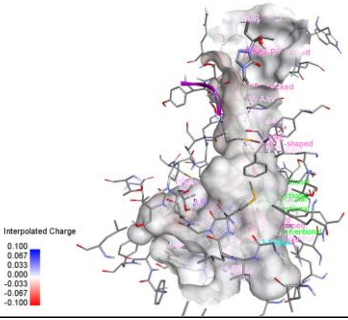	**Tocopherol** 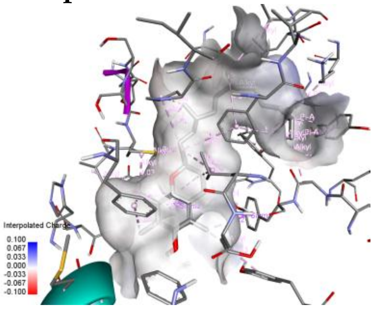	**Kaempferol** 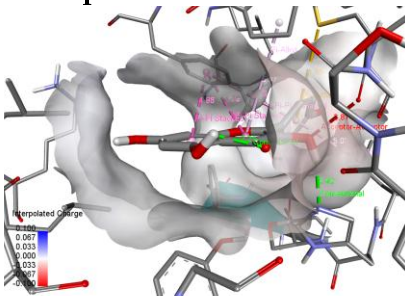
**Cadinol**	**Elemol**	**Thyme-2**

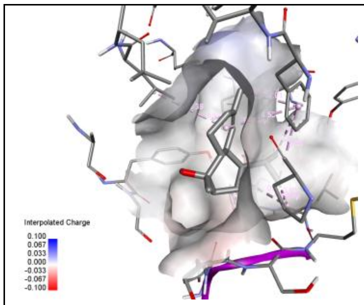

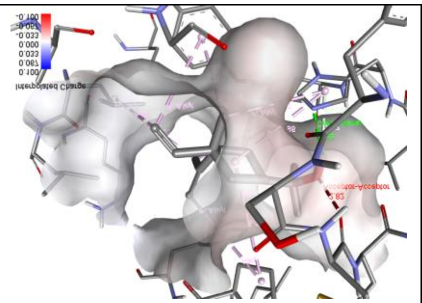

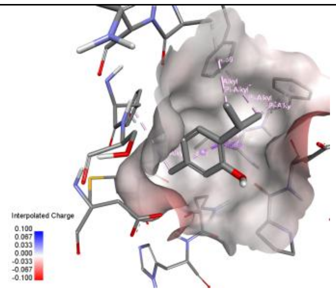

Thyme-3

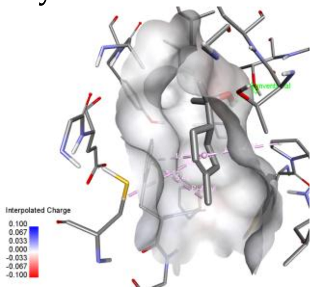

Thyme-5

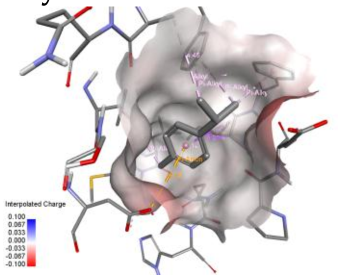

Thyme-1

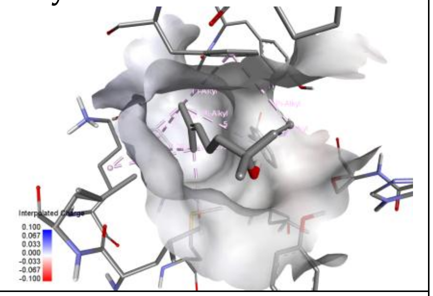

Thyme-4

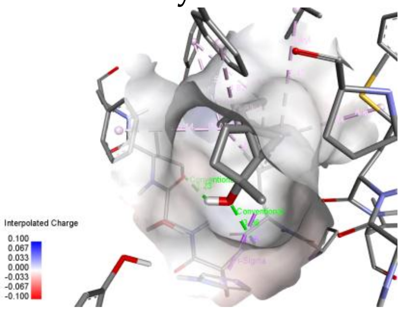

Pinene

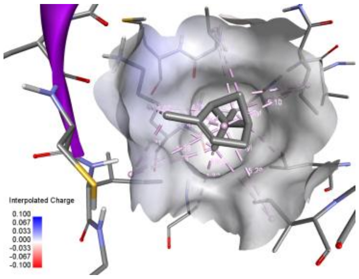

Cryptone

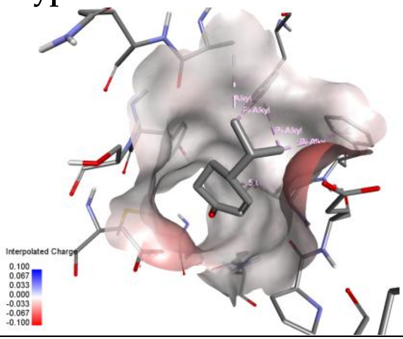

Glutamine

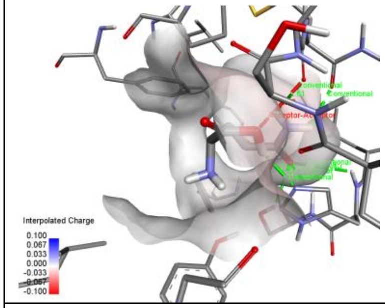

Figure S4: Charges formation between the proposed molecules and Sterol 14-alpha demethylase.

**Posaconazole** 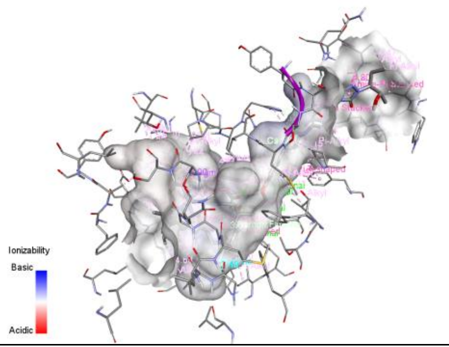	**Tocopherol** 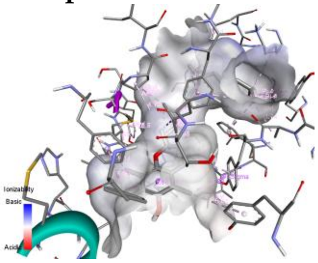	**Kaempferol** 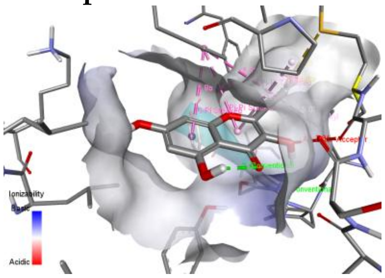
**Cadinol** 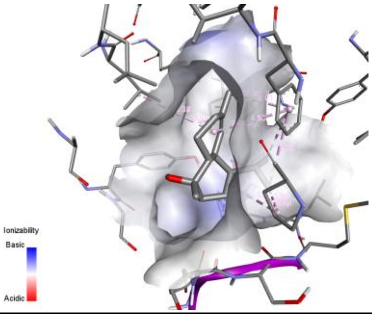	**Elemol** 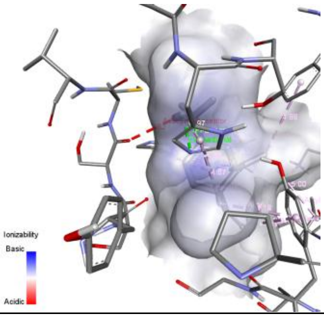	**Thyme-2** 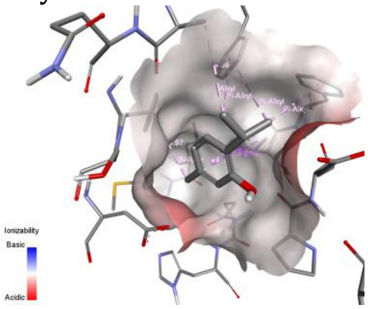
**Thyme-3** 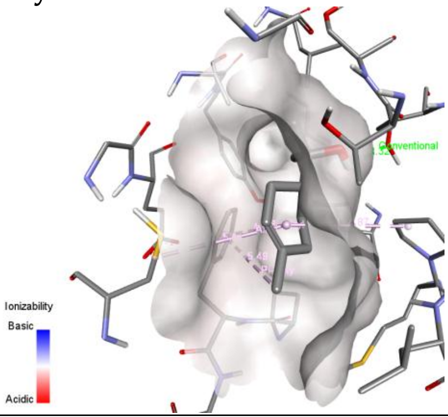	**Thyme-5** 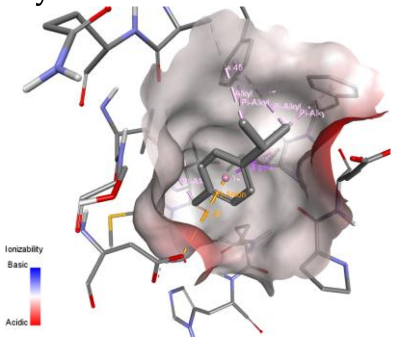	**Thyme-1** 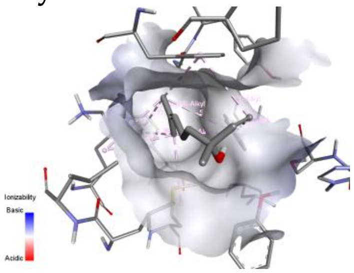
**Thyme-4** 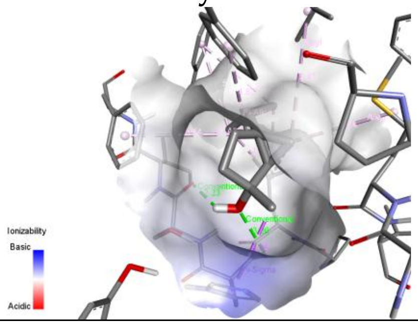	**Pinene** 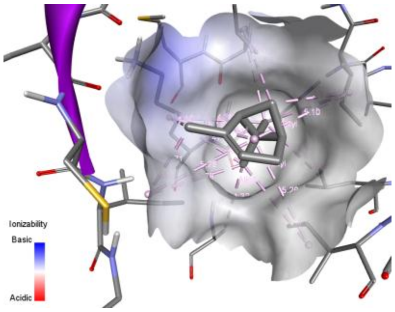	**Cryptone** 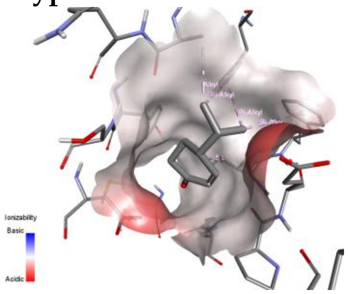
**Glutamine**		

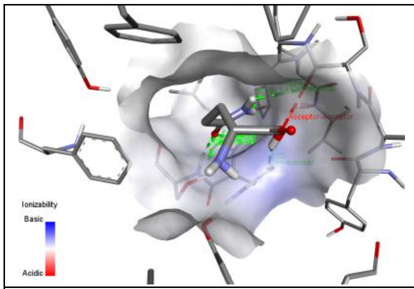		

Figure S5: Ionizability of the interfaces between the proposed molecules and Sterol 14-alpha demethylase.

**HQQ** 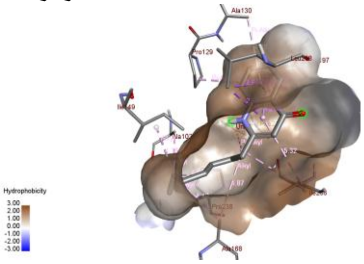	**Tocopherol** 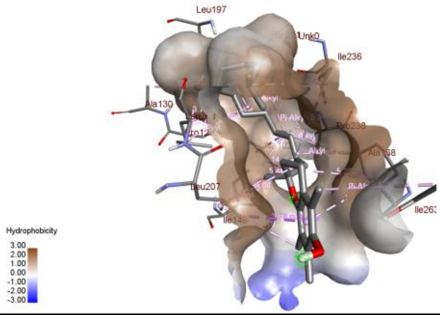	**Kaempferol** 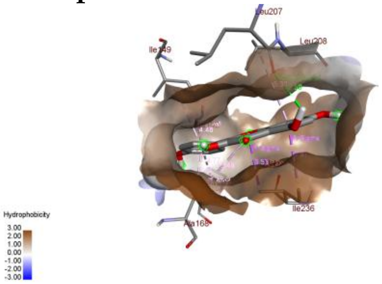
**Cadinol** 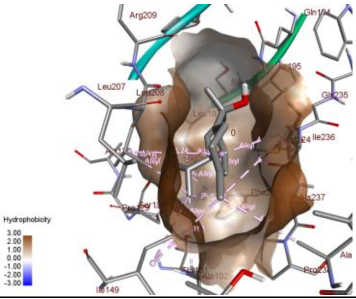	**Thyme-2** 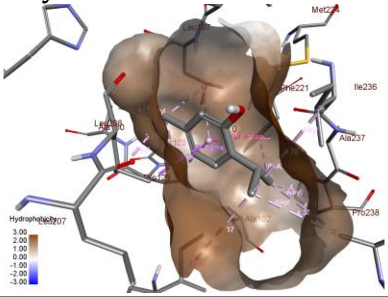	**Cryptone** 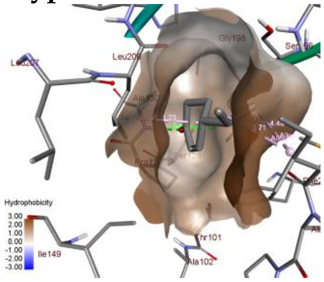
**Thyme-5** 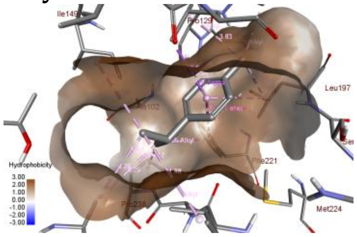	**Thyme-3** 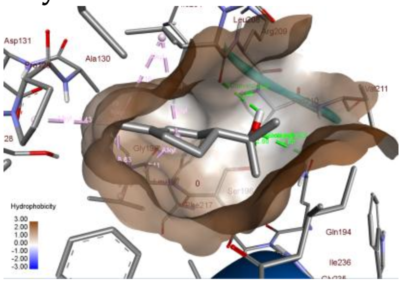	**Elemol** 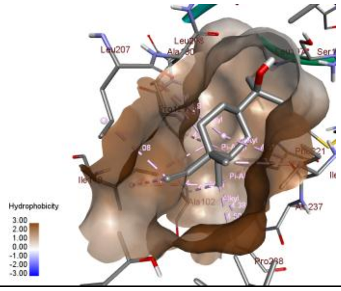
**Thyme-4**	**Pinene** 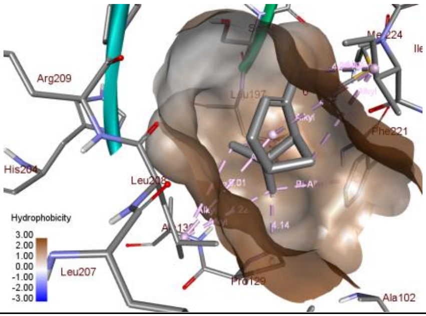	**Thyme-1**

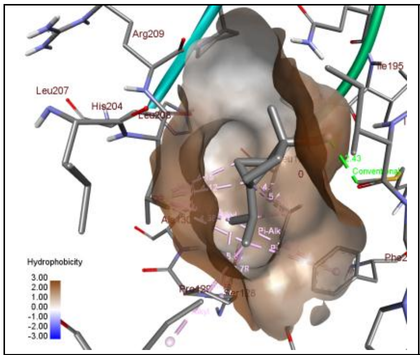		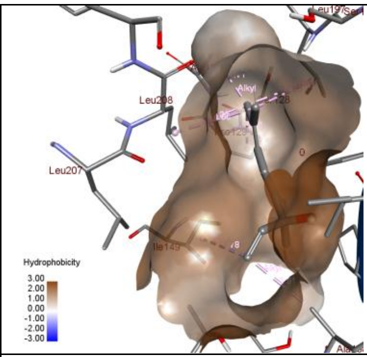
**Glutamine** 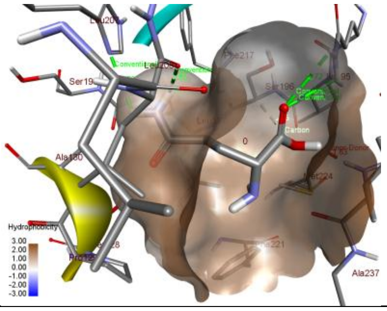		
**Figure S6:** Hydrophobic interfaces involved in the interaction of the proposed molecules with MvfR.		

**HQQ** 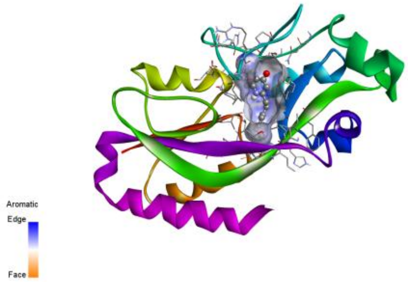	**Tocopherol** 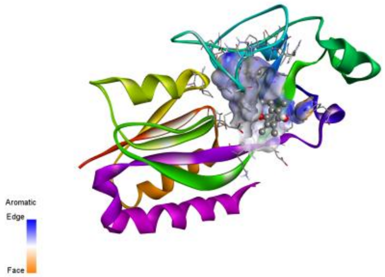	**Kaempferol** 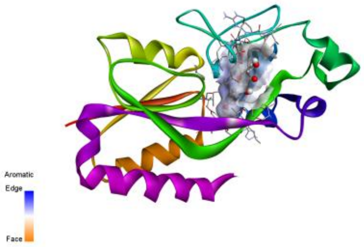
**Cadinol** 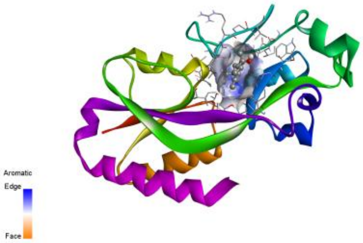	**Thyme-2** 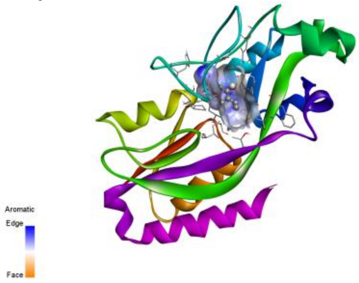	**Cryptone** 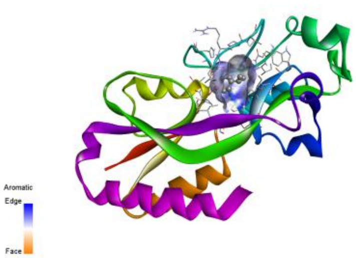
**Thyme-5**	**Thyme-3**	**Elemol**

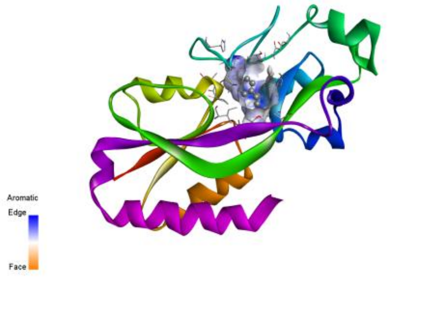	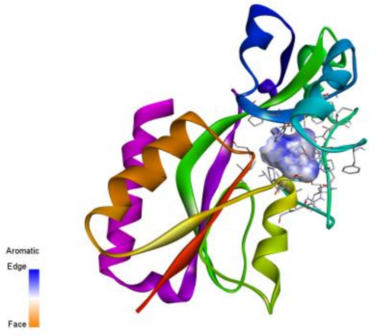	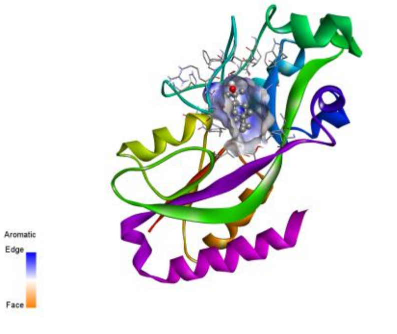
**Thyme-4**<br>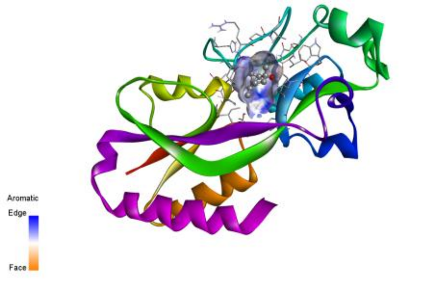	**Pinene**<br>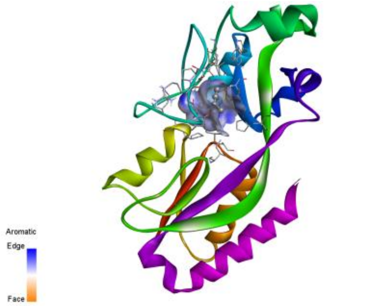	**Thyme-1**<br>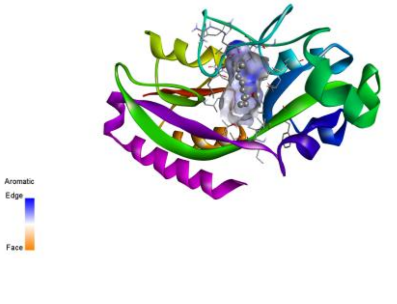
**Glutamine**<br>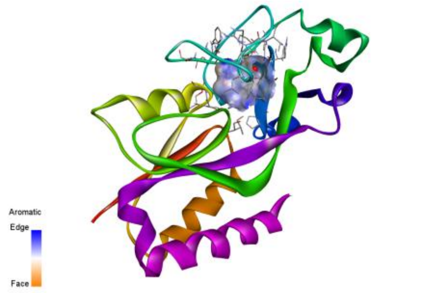		

Figure 7: Aromatic surface view of the proposed molecules on MvfR.

**HQQ**<br>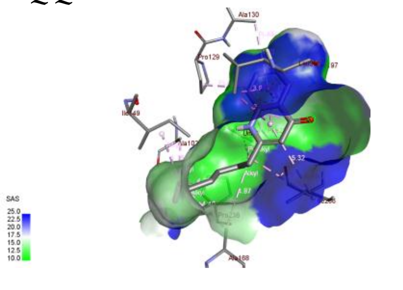	**Tocopherol**<br>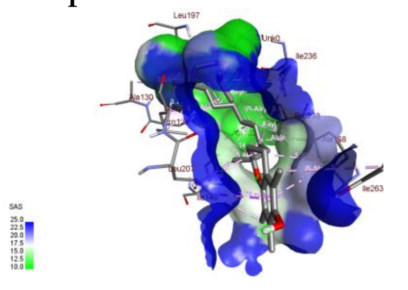	**Kaempferol**<br>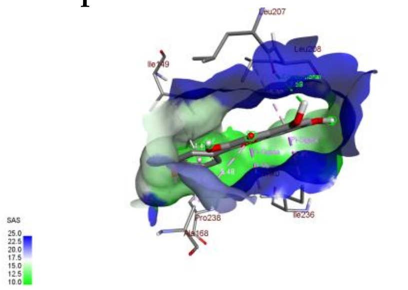
**Cadinol**	**Thyme-2**	**Cryptone**

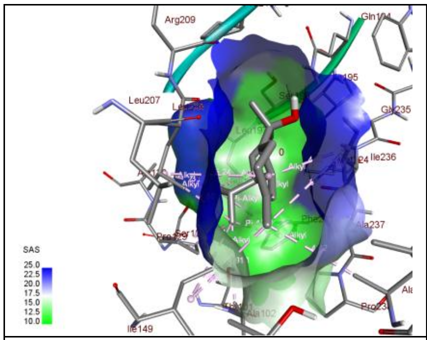

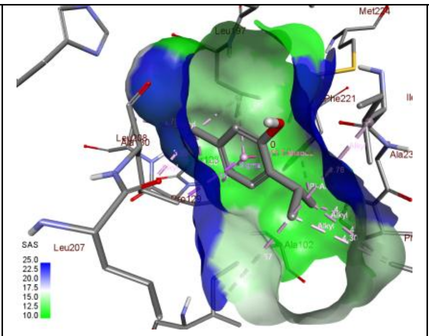

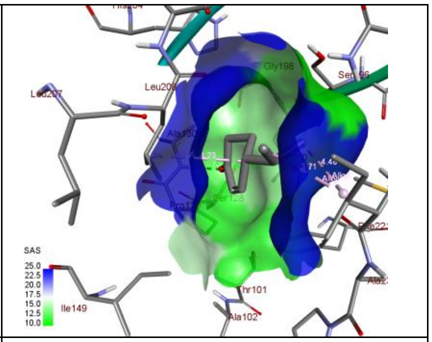

Thyme-5

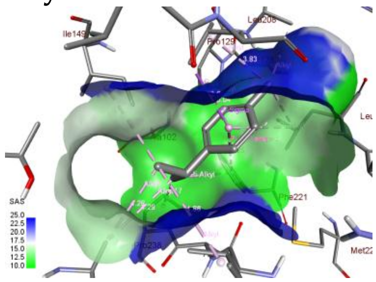

Thyme-3

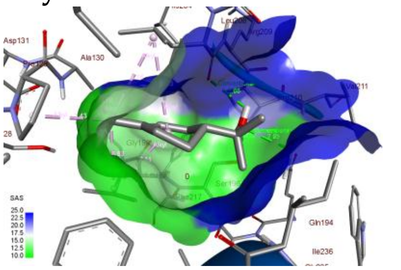

Elemol

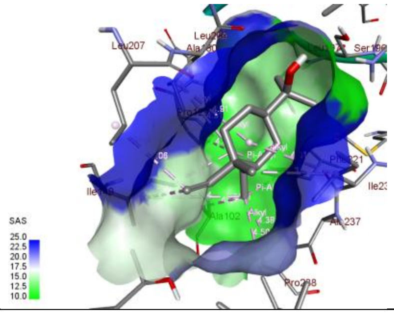

Thyme-4

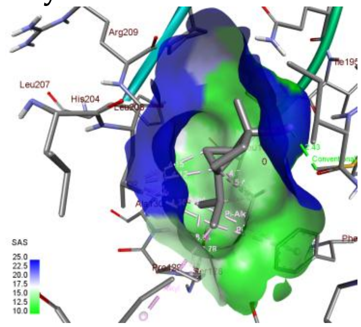

Pinene

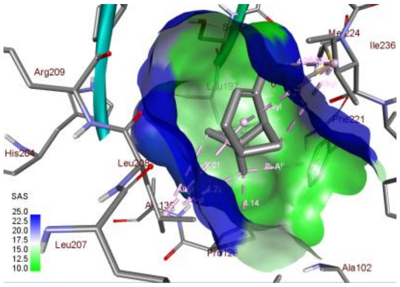

Thyme-1

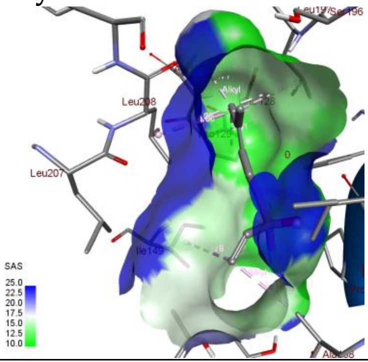

Glutamine

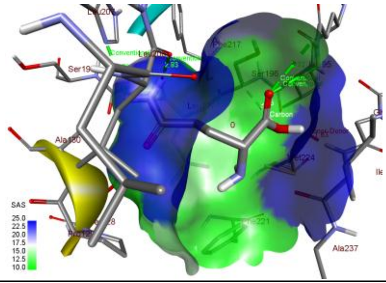

Figure S8: Solvent-accessible area on the interface between of the ligands and MvfR.

**HQQ**<br>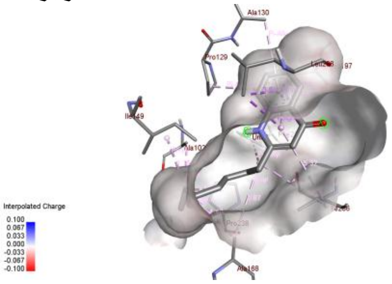	**Tocopherol**<br>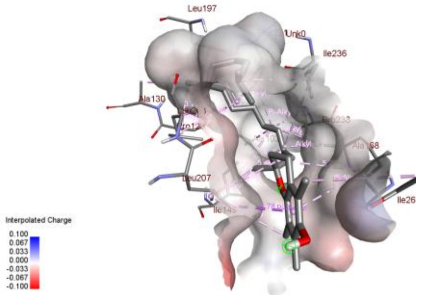	**Kaempferol**<br>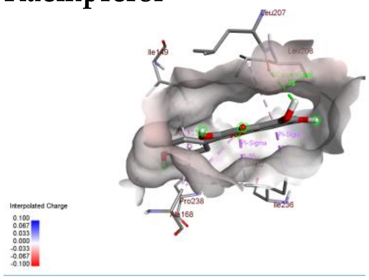
**Cadinol**<br>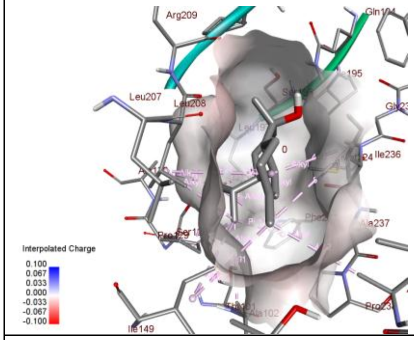	**Thyme-2**<br>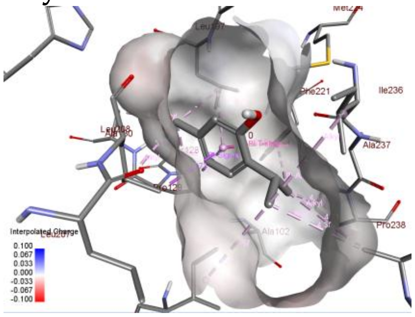	**Cryptone**<br>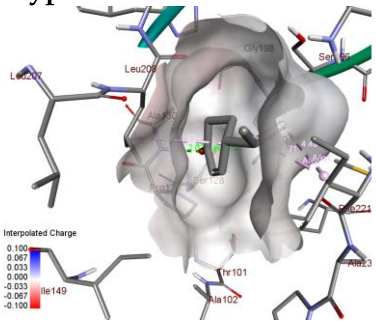
**Thyme-5**<br>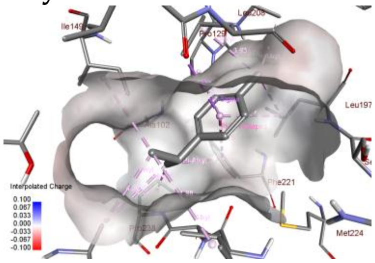	**Thyme-3**<br>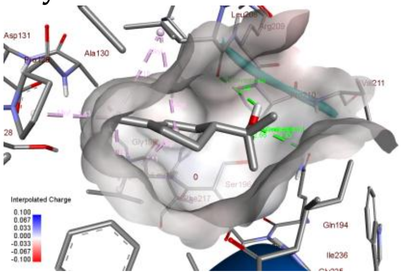	**Elemol**<br>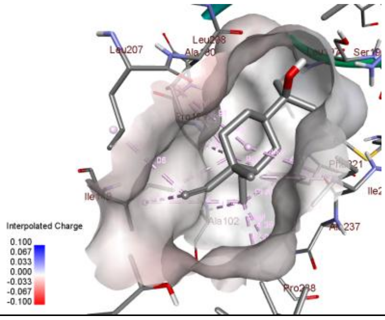
**Thyme-4**<br>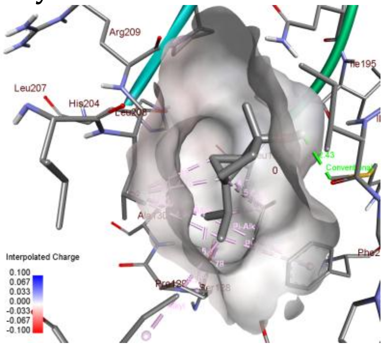	**Pinene**<br>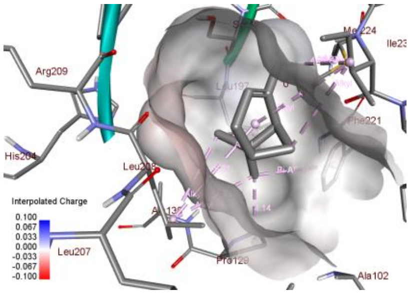	**Thyme-1**<br>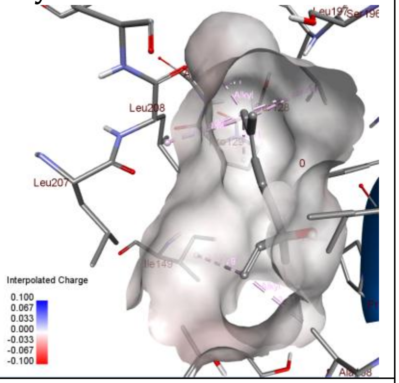
**Glutamine**		

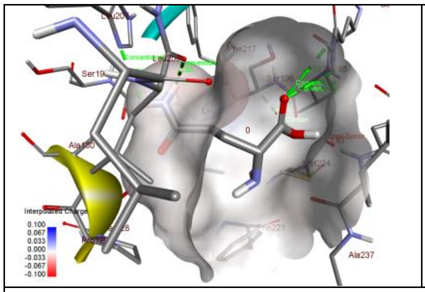		
**Figure S9:** Charges formation between the proposed molecules and MvfR.		

**HQQ** 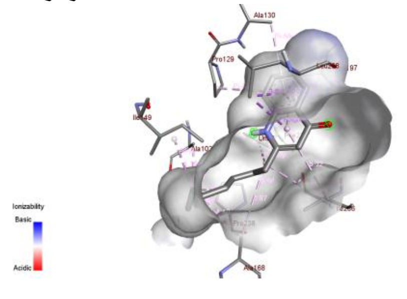	**Tocopherol** 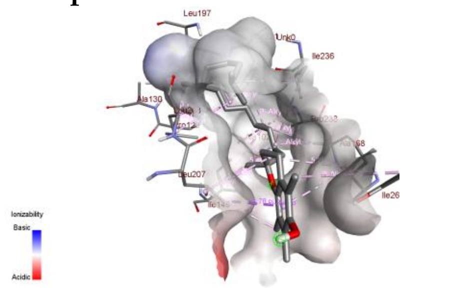	**Kaempferol** 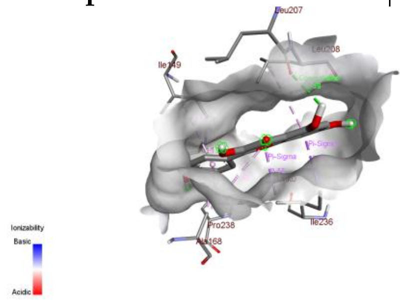
**Cadinol** 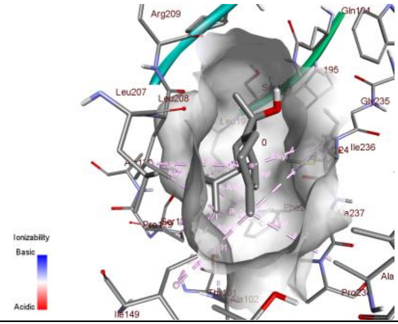	**Thyme-2** 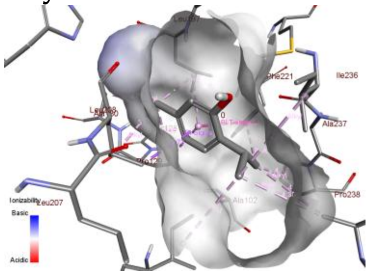	**Cryptone** 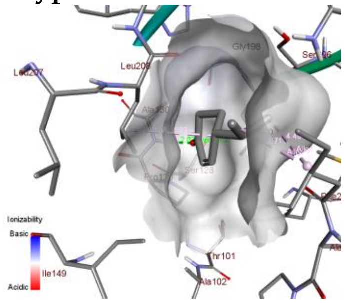
**Thyme-5** 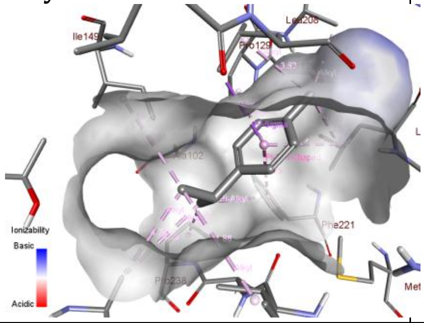	**Thyme-3** 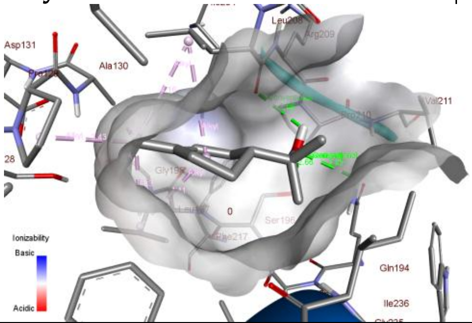	**Elemol** 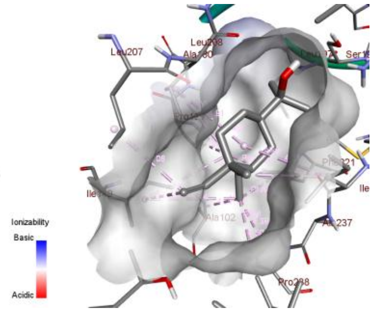
**Thyme-4**	**Pinene** 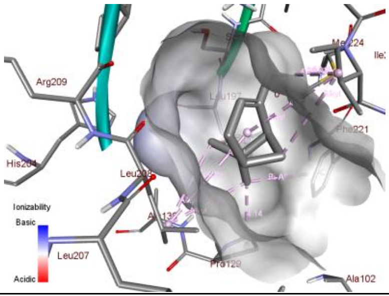	**Thyme-1**

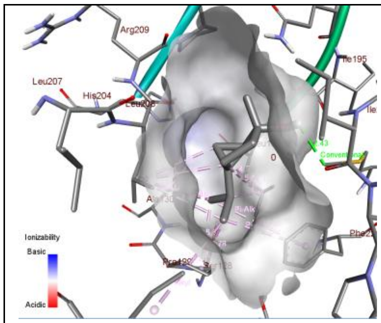 		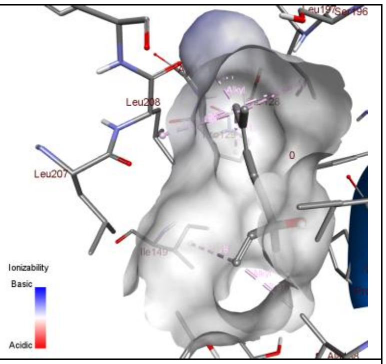
**Glutamine**<br>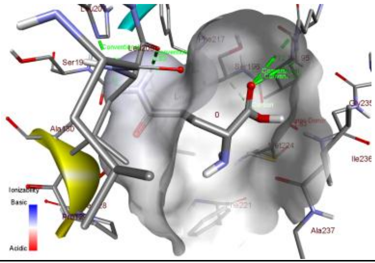 		

Figure S10: Ionizability of the interfaces between the proposed molecules and MvfR.
